# Framework disorder and its effect on selective hysteretic sorption of a T-shaped azole-based metal–organic framework

**DOI:** 10.1107/S2052252518015749

**Published:** 2019-01-01

**Authors:** Sujuan Wang, Zhang-Wen Wei, Jianyong Zhang, Long Jiang, Dingxin Liu, Ji-Jun Jiang, Rui Si, Cheng-Yong Su

**Affiliations:** a MOE Laboratory of Bioinorganic and Synthetic Chemistry, Lehn Institute of Functional Materials, Sun Yat-Sen University, Guangzhou 510275, People’s Republic of China; b Shanghai Institute of Applied Physics, Chinese Academy Sciences, Shanghai Synchrotron Radiation Facility, Shanghai 201204, People’s Republic of China

**Keywords:** metal–organic frameworks, T-shaped ligands, disorder, MOFs, solid properties, channel structures, gas separation

## Abstract

Metal–organic frameworks with highly ordered porosity have been studied extensively. In this paper, the effect of framework (pore) disorder on the gas sorption of azole-based isoreticular Cu(II) MOFs with **rtl** topology and characteristic 1D tubular pore channels is investigated for the first time. In contrast to other isoreticular **rtl** metal–organic frameworks, the Cu(II) metal–organic framework based on 5-(1*H*-imidazol-1-yl)isophthalate acid has a crystallographically identifiable disordered framework without open N-donor sites. The framework provides a unique example for investigating the effect of pore disorder on gas sorption that can be systematically evaluated. It exhibits remarkable temperature-dependent hysteretic CO_2_ sorption up to room temperature, and shows selectivity of CO_2_ over H_2_, CH_4_ and N_2_ at ambient temperature. The unique property of the framework is its disordered structure featuring distorted 1D tubular channels and DMF-guest-remediated defects. The results imply that structural disorder (defects) may play an important role in the modification of the performance of the material.

## Abstract 2

The effect of framework (pore) disorder on gas sorption of azole-based isoreticular Cu(II) MOFs with **rtl** (rutile) topology and characteristic 1D tubular pore channels is investigated for the first time.

## 1. Introduction   

Porous materials such as zeolites, molecular cages, activated carbons, covalent organic frameworks and metal–organic frameworks (MOFs) have been utilized as adsorbents for gas capture (*e.g.* CO_2_). Among these materials, MOFs with high surface area, tunable pore size and modifiable pore surfaces are receiving great interest because they show unique flexibility and dynamic behaviors (*e.g.* phase change) and also offer significant improvements in separation performance (Horike *et al.*, 2009 ▸; Schneemann *et al.*, 2014 ▸; Alhamami *et al.*, 2014 ▸; Zhang *et al.*, 2017 ▸). For example, their corresponding hysteretic gas adsorption/desorption behaviors are able to decrease the pressure of gas storage.

According to the isoreticular MOF concept introduced by Yaghi and others (Eddaoudi *et al.*, 2002 ▸; Wilmer *et al.*, 2012 ▸; Deng *et al.*, 2012 ▸; Ma *et al.*, 2010 ▸), a MOF structure is determined by the connectivity of the rigid bridging ligand, the secondary building unit (SBU) and the framework topology. By fixing a specific topology, the isostructural frameworks can be readily fine-tuned via organic ligand manipulation and metal ion selection (Yuan *et al.*, 2010 ▸; Caskey *et al.*, 2008 ▸). Analogous ligands with different functionalities can be especially employed to produce desired frameworks (Colombo *et al.*, 2012 ▸; Sumida *et al.*, 2013 ▸). Along these lines, the effect of some subtle factors on the pore properties can be investigated and analyzed by designing isoreticular MOFs (McDonald *et al.*, 2012 ▸; Li, Zhang *et al.*, 2012 ▸; Das *et al.*, 2012 ▸; Zhang *et al.*, 2012 ▸; Bae *et al.*, 2012 ▸). However, much of the research emphasis remains on developing and characterizing highly ordered framework materials with regularly repeating crystal structures where all the pores have exactly the same size, shape and functionality. This ignores disorder and/or defects which are often concomitant with the growth of a long-range periodic framework. The introduction of defects may alter the regular porous interior and behavior of MOFs. Defects may play a crucial role in enhanced accessibility of the porous network and higher basicity of the metal centers. Defects in regular MOFs have been shown to impart unusual and useful physical properties to these framework materials (Cairns & Goodwin, 2013 ▸; Tucker *et al.*, 2005 ▸; Goodwin *et al.*, 2009 ▸; Cheetham *et al.*, 2016 ▸; Fang *et al.*, 2015 ▸; Tahier & Oliver, 2017 ▸; Cliffe *et al.*, 2014 ▸; Li *et al.*, 2013 ▸). Fundamental correlations between defects and properties of the resulting defective materials have been shown in some examples. Partial interpenetration of MOF NOTT-202a was observed by Schröder and co-workers to provide defect sites for gas recognition and storage, and to show selective hysteretic sorption of gas molecules (Yang *et al.*, 2012 ▸, 2013 ▸). The mixed linker approach was reported by Zhou and co-workers to introduce functionalized disordered mesopores into MOFs (Park *et al.*, 2012 ▸; Yuan *et al.*, 2016 ▸). Missing-linker defects were shown to be extensively present [*e.g.* in the UiO-66 series (Trickett *et al.*, 2015 ▸)], increasing the gas adsorption capacities (Rodríguez-Albelo *et al.*, 2017 ▸) and enhancing the proton mobility (Taylor, Dekura *et al.*, 2015 ▸; Taylor, Komatsu *et al.*, 2015 ▸). Herein, we report a unique example to demonstrate how structural disorder can lead to unusual changes in the properties of MOFs (*e.g.* the sorption property) based on a series of isoreticular azole-based MOFs with **rtl** (rutile) topology (denoted **rtl**-MOFs).

**Figure 1:**
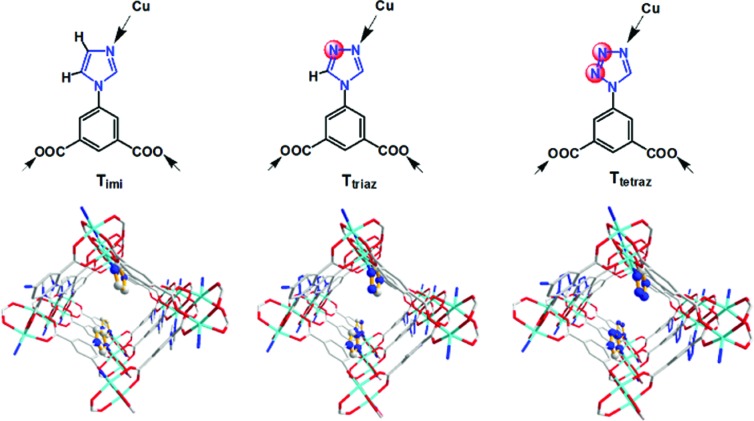
T-shaped bridging ligands containing one azole five-membered-ring heterocycle (imidazole, 1,2,4-triazole and tetrazole) and two carboxylate groups, along with the X-ray crystal structure comparisons of the azole-based **rtl**-MOFs (T_imi_-Cu, T_triaz_-Cu and T_tetraz_-Cu) showing variation of N sites along the Cu_8_ *L*_4_ squares on the inner pore surface.

Isoreticular **rtl**-MOFs are based on the square paddle-wheel SBU, one of the most common SBUs formed by metals and carboxyl­ates (Tranchemontagne *et al.*, 2009 ▸). Such square SBUs have long been used to build various porous structures, for example, pillared layer structures with mixed ligands of N-containing heterocycles (*e.g.* pyridine, imidazole, pyrazole, tetrazole, 1,2,4-triazole) and carboxyl­ate compounds (Seki & Mori, 2002 ▸; Dybtsev *et al.*, 2004 ▸; Pichon *et al.*, 2007 ▸; Chen, Fronczek, Courtney *et al.*, 2006 ▸; Chen, Ma, Zapata *et al.*, 2006 ▸, 2007 ▸). We and others have implemented a new pillaring strategy (Suh *et al.*, 2012 ▸) by amalgamation of one heterocycle and two carboxylate groups into a T-shaped heterofunctional ligand, resulting in the pillaring of 2D-edge transitive nets by 3-connected nodes (Xiang *et al.*, 2011 ▸; Eubank *et al.*, 2011 ▸; Wen *et al.*, 2012 ▸; Zhang *et al.*, 2010 ▸; Chen *et al.*, 2011 ▸, 2015 ▸; Jia *et al.*, 2011 ▸; Du *et al.*, 2013 ▸; Kobalz *et al.*, 2016 ▸; Cheng *et al.*, 2017 ▸; Wei *et al.*, 2014 ▸). Therefore, based on the square-grid **sql** layer (Zou *et al.*, 2007 ▸; Zhong *et al.*, 2011 ▸; Xue *et al.*, 2007 ▸; Bourne *et al.*, 2001 ▸; Gao *et al.*, 2003 ▸) formed from the isophthalate unit, a series of isoreticular MOFs have been developed (Xiang *et al.*, 2011 ▸; Eubank *et al.*, 2011 ▸; Wen *et al.*, 2012 ▸; Zhang *et al.*, 2010 ▸; Chen *et al.*, 2011 ▸, 2015 ▸; Jia *et al.*, 2011 ▸; Du *et al.*, 2013 ▸; Kobalz *et al.*, 2016 ▸; Cheng *et al.*, 2017 ▸). These microporous MOFs feature in (3,6)-connected 3D frameworks displaying **rtl** topology and point (Schläfli) symbol (4·6^2^)_2_(4^2^·6^10^·8^3^). The T-shaped ligand serves as a 3-connected node while the paddle-wheel cluster acts as a 6-connected node. A variety of heterocycles [pyridine (Xiang *et al.*, 2011 ▸; Eubank *et al.*, 2011 ▸; Chen *et al.*, 2015 ▸), pyrimidine (Du *et al.*, 2013 ▸), 1,2,4-triazole (Eubank *et al.*, 2011 ▸; Wen *et al.*, 2012 ▸; Kobalz *et al.*, 2016 ▸) and tetrazole (Zhang *et al.*, 2010 ▸)] have been incorporated into the T-shaped ligands and various metal ions [Cu (Xiang *et al.*, 2011 ▸; Eubank *et al.*, 2011 ▸; Wen *et al.*, 2012 ▸; Zhang *et al.*, 2010 ▸; Du *et al.*, 2013 ▸; Kobalz *et al.*, 2016 ▸; Chen *et al.*, 2011 ▸), Zn (Chen *et al.*, 2011 ▸) and Co (Jia *et al.*, 2011 ▸)] have been utilized to form square paddle-wheel SBUs. One unique characteristic of the **rtl** topology is that such frameworks are forbidden from interpenetration, so that 1D tubular channels can be formed and isolated by the walls of the parallel pillars containing the aromatic rings. On one hand, the pore aperture is largely defined by the **sql** grid size or the distance between two carboxylate groups of the isophthalate unit. Thus, the pore sizes are similar for these isoreticular MOFs. On the other hand, the pore surface can be modified by changing or functionalizing the heterocyclic rings [*e.g.* with uncoordinated N atoms (Eubank *et al.*, 2011 ▸) or alkyl groups (Kobalz *et al.*, 2016 ▸; Cheng *et al.*, 2017 ▸)], which significantly enhance the gas selectivity. In this paper, we add a unique member with framework (pore) disorder into an isoreticular **rtl**-MOF by incorporating an imidazole ring into the T-shaped ligand (Fig. 1 ▸). The remarkable effect of framework (pore) disorder on the sorption property, which induces a significant hysteretic sorption for CO_2_ at room temperature, is investigated.

## 2. Results and discussion   

### 2.1. Syntheses and crystal structures   

The T-shaped ligand 5-(1*H*-imidazol-1-yl)isophthalate acid (denoted as T_imi_) used to introduce hetereocyclic imidazole was synthesized by acid-catalyzed ester hydrolysis of dimethyl 5-(1*H*-imidazol-1-yl)isophthalate, which was obtained via cyclization of formaldehyde with the diazabutadiene intermediate formed from a reaction of molar equivalents of di­methyl 5-amino­isophthalate, ammonium chloride and glyoxal. A mild solvothermal reaction of T_imi_ with CuCl_2_·2H_2_O at 353 K in a mixture of DMF/EtOH (*v*:*v* 3:1) led to the new **rtl**-MOF member, herein denoted as T_imi_-Cu.

**Figure 2:**
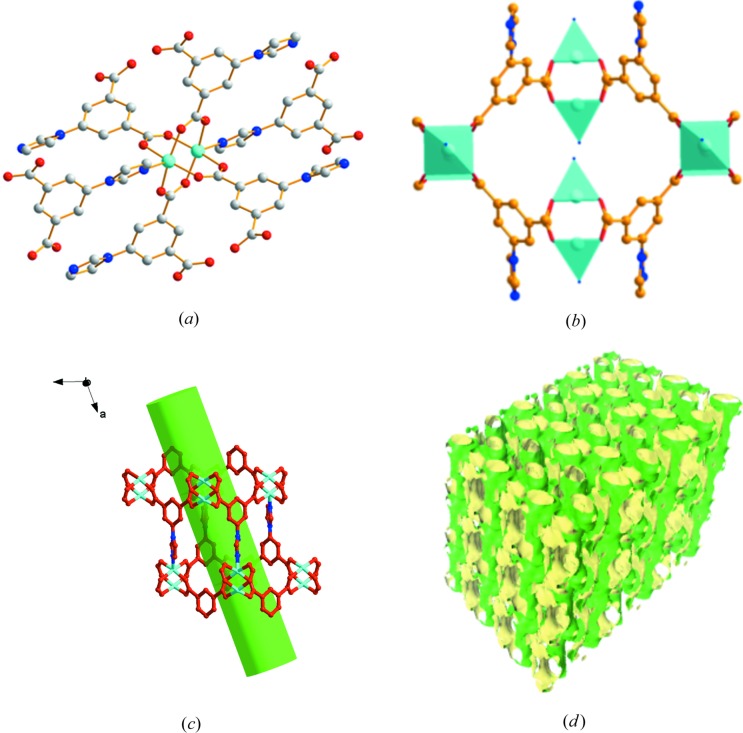
X-ray crystal structures of T_imi_-Cu: (*a*) Cu_2_ paddle-wheel SBU, (*b*) Cu_8_ *L*_4_ square in **sql** layer formed via the isophthalate moiety in a 1,2-alternate fashion (up–up down–down), (*c*) side view of the 1D channel formed via pillared Cu_8_ *L*_4_ square-grid **sql** layers, (*d*) solvent-accessible voids in the tubular channels perpendicular to the *bc* plane. Framework disorder, solvated molecules and hydrogen atoms have been omitted for clarity.

The structure of T_imi_-Cu was determined and checked by single-crystal X-ray diffraction at 150, 195 and 273 K with several randomly selected crystals, all displaying disordered character. In general, T_imi_-Cu crystallizes in the monoclinic space group *P*2_1_/*c*, which is isostructural to the previously reported T_triaz_-Cu (Eubank *et al.*, 2011 ▸) and T_tetraz_-Cu (Zhang *et al.*, 2010 ▸) (Fig. 1 ▸, Table S1 of the supporting information). In contrast, the reaction of T_imi_ with CuCl_2_·2H_2_O at 353 K in acidified DMF yields a different topological structure (Zhu *et al.*, 2015 ▸). The reaction of T_imi_ with CuBr_2_ at 353 K in acidified DMF–H_2_O yields the same topological structure, but no framework disorder is located (this structure is not stable during a sorption study) (Cheng *et al.*, 2017 ▸). Therefore, regardless of the disorder in T_imi_-Cu, these azole-based **rtl**-MOFs have the same overall unit cell, building block geometry and lattice porosity. As shown in Fig. 2 ▸, the basic structural units are the dicopper paddle-wheel SBUs bonded together by four T-shaped ligands via carboxylate groups. The axial sites are occupied by the N donors of imdazole. Each Cu_2_ SBU joins six ligands and each ligand bridges three different Cu_2_ SUBs, thus generating the (3,6)-connected 3D framework of **rtl** topology. The tubular channels run along the *a* axis and have an opening of 11.9 × 14.5 Å (*b* × *c*, *b* ⊥ *c*) along the diagonals of the quadrangle cross section. The solvated DMF and H_2_O molecules are disordered and reside in the channels. Based on calculations using the program *PLATON*, the total potential solvent-accessible void volume is about 885.1 Å^3^ per unit cell with a pore volume ratio of 50.3%.

**Figure 3:**
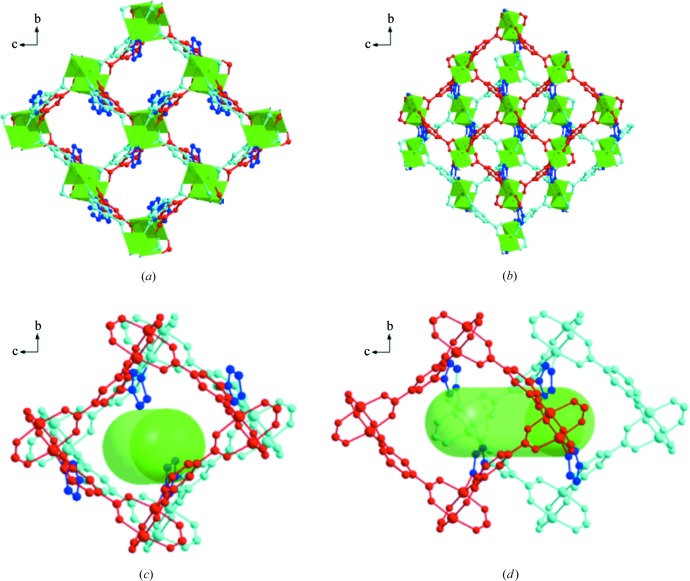
Comparison of the ideally ordered framework and one possible local disordered framework of T_imi_-Cu in the *a* (upper) and *b* (lower) directions: (*a*) ordered overlapping of the Cu_8_ *L*_4_ square-grid **sql** layers, (*b*) one possible disordered offsetting model of the Cu_8_ *L*_4_ square-grid **sql** layers, (*c*) top view of 1D straight channel in the ordered framework formed via overlapping of Cu_8_ *L*_4_ square grids and (*d*) top view of the 1D distorted channel in the disordered framework formed via offsetting of the Cu_8_ *L*_4_ square grid.

**Figure 4:**
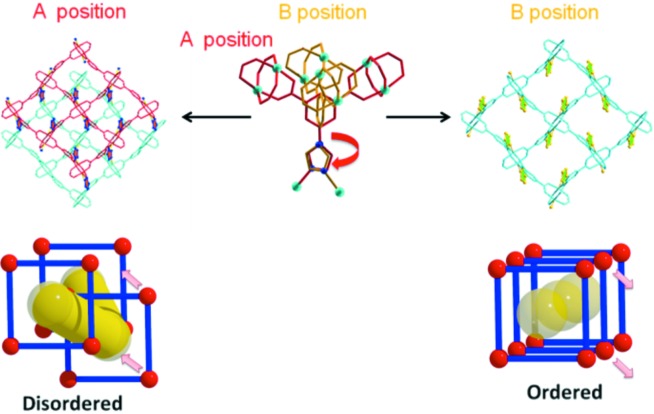
Disordered framework versus ordered framework for T_imi_-Cu.

The detailed structural analysis of T_imi_-Cu reveals that the coordination framework is actually disordered in a specific fashion (Figs. 3 ▸ and S1). In a statistical sense, each Cu_2_ paddle-wheel SBU is distributed over two fractional positions in an approximate 3:1 ratio, and some local areas can be imagined to superimpose two partial **rtl**-MOFs in an offset way. In reality, each Cu_2_ SBU has a definite orientation in the individual asymmetric unit, and is closely related to the neighboring SBUs owing to the rigidity of the isophthalate moiety and consequently fixed Cu_8_ *L*_4_ square-grid conformation. This is due to the fact that, in the **rtl** topology, **sql** sheets are formed via the isophthalate moiety in a predetermined 1,2-alternating fashion (up–up down–down). Thus, local primary 2D Cu_8_ *L*_4_ square-grid layers are inherently inert to dynamic disorder. In the contrast, local interlayer gliding is relatively easy (Fig. 4 ▸) because: (i) the crystal structure is stacked with the parallel 2D **sql** square grids via pillars of the T-shaped T_imi_ ligands, (ii) the Cu—N binding between imidazole N donors and axial positions of Cu_2_ SBUs is relatively labile, and (iii) the imidazole ring is freely rotatable along the N—C bond to the isophthalate moiety so as to adapt to the layer motions. Therefore, the disorder in T_imi_-Cu may be considered to originate from local random interpolated movement of the 2D Cu_8_ *L*_4_ square-grid layers. This might also account for the observation of two isomerized T_triaz_-Cu structures (Eubank *et al.*, 2011 ▸; Wen *et al.*, 2012 ▸) which differ only in the arrangement of the 2D Cu_8_ *L*_4_ square-grid layers in one direction. The crystal packing in the other two directions and the framework porosity are similar. However, as for T_teraz_-Cu, noticeable disorder was not detected in T_triaz_-Cu, meaning that the chemical nature of heterocycles in this azole-based isoreticular **rtl**-MOF has a subtle influence on crystallization habit and framework isomerization (Makal *et al.*, 2011 ▸; Lü *et al.*, 2006 ▸). In the reaction of T_imi_ with CuBr_2_ at 353 K in acidified DMF–H_2_O, the product has no noticeable disorder as well, showing that guest solvent molecules or ions may have a subtle effect on the disorder.

To further probe the structure of T_imi_-Cu, X-ray photoelectron spectroscopy (XPS, Fig. S2) and X-ray absorption fine-structure (XAFS, Fig. S3 and Table S2) measurements were investigated. XPS confirms the presence of copper(II) in T_imi_-Cu. Cu 2*p* core-level photoelectron spectra for imi-Cu displayed doublets *i.e.* Cu 2*p*_3/2_ and Cu 2*p*_1/2_ at 935.8 and 955.0 eV, respectively. The Cu 2*p*_3/2_ and Cu 2*p*_1/2_ main doublets were separated by 20 eV and their satellite peaks present at binding energies of 943.0 and 964.0 eV are characteristic of the unfilled orbitals. The study of the Cu LMM signal also supports the presence of Cu(II), and the Cu LMM signal with a kinetic energy of 916.0 eV is assigned to Cu(II). Although being the most common technique for structure determination/identification and measurement of long-range order, powder X-ray diffraction (PXRD) generally provides little information on defects. XAFS was expected to provide some local order information. XAFS data show that Cu(II) is present and the average Cu—O/N bonds are calculated to be 1.97 ± 0.01 Å. However, the data of T_imi_-Cu are comparable with the data of Cu-T_triaz_ and Cu-T_tetraz_, which have no disorder. It can be concluded that T_imi_-Cu and the other two MOF materials have similar short-range coordination spheres.

The above results show that a unique structural feature of T_imi_-Cu is that the disorder does not cause absence of crystallinity. In other words, the disorder occurs in a crystallographically ‘regular’ way to a certain extent. Various comparisons of the disordered structures of T_imi_-Cu with those of notionally ordered counterparts are depicted in Figs. 3 ▸, 4 and S1. It is speculated that partial Cu_8_ *L*_4_ square-grid layers shift exactly half a unit cell along the *c* direction, leading to offset stacking of these 2D **sql** sheets along the *a* direction. In this way, all Cu_2_ SBUs need not change conformation except for a 180° rotation of the imdazole rings to adapt to the new axial coordination (Fig. 4 ▸). Since orientation of the Cu_2_ SBUs in every layer is fixed, gliding of the 2D sheet along any other direction will cause a severe geometry and connectivity mismatch. This is evident from observations of the nearly identical packing modes of the ordered and disordered frameworks in both the *b* and *c* directions (Fig. S1). Such a disorder phenomenon is distinct from the topological disorder in well known amorphous silica glass (α-SiO_2_) with a continuous random network (Cairns & Goodwin, 2013 ▸; Tucker *et al.*, 2005 ▸). It is also distinct from the 2D layered square grids Ni(CN)_2_, which lack long-range order in the perpendicular direction (Cairns & Goodwin, 2013 ▸; Goodwin *et al.*, 2009 ▸), although in a short-range or local environment they might be comparable. It is worth noting that some framework defects like coordination mismatch or connectivity distortion should also be present.

Therefore, the disordered T_imi_-Cu is a new member of the azole-based isoreticular **rtl**-MOFs containing different five-membered-ring heterocycles (imidazole, 1,2,4-triazole and tetrazole). A study of T_imi_-Cu may offer the following advantages: (i) T_imi_-Cu has a comparable pore volume regardless of chemical or structural variation. For the azole-based **rtl**-MOFs, the heterocycles protrude into the channels and slightly reduce the pore sizes in comparison with the pyridyl **rtl**-MOF (Xiang *et al.*, 2011 ▸). However, the similar Cu_8_ *L*_4_ square grids and pillaring nature of three five-membered azole rings principally decide the total comparable pore volume. (ii) The inner pore surfaces are modified by different heterocyclic azole rings. Two C—H moieties point into the channel in T_imi_-Cu, one C—H and one N donor in T_triaz_-Cu, while there are two N donors in T_tetraz_-Cu (Fig. 1 ▸). (iii) Framework disorder in T_imi_-Cu produces different pore permeability from that in T_triaz_-Cu and T_tetraz_-Cu. As seen from Figs. 3 ▸, 4 and S1, partial gliding of **sql** sheets does not cause significant pore changes along the *b* and *c* axes but does along the *a* axis. The main pore channels along the *a* axis in T_imi_-Cu become distorted in contrast to the straight channels with long-range order in T_triaz_-Cu and T_tetraz_-Cu. (iv) The propensity for disorder and framework defects in T_imi_-Cu probably results in sorption dynamics compared with the more static frameworks in T_triaz_-Cu and T_tetraz_-Cu.

### 2.2. Thermal stability   

As-synthesized bulk samples of T_imi_-Cu display sharp PXRD patterns that closely resemble those simulated from the single-crystal data (Fig. S4), indicative of phase purity and air stability. Thermogravimetric analysis (TGA) of T_imi_-Cu reveals similar thermal stability to that reported for T_tetraz_-Cu (Wen *et al.*, 2012 ▸; Zhang *et al.*, 2010 ▸). The TGA plot of as-synthesized T_imi_-Cu shows a weight loss of 26.2% from room temperature to *ca*. 513 K, corresponding to the release of solvent molecules (1.5 DMF and 0.5 H_2_O per formula unit; calculated weight loss 28.8%) residing in the pore channels (Fig. S5). Rapid decomposition occurs upon further heating above 543 K. The thermostability of the desolvated material is further revealed by PXRD experiments at various temperatures (Fig. S6). PXRD results reveal that the framework structure is unchanged up to 373 K and transforms into a new structure with a different framework topology between 383 and 443 K (the new structure will be reported in due course). This further indicates that the Cu^2+^-T_imi_ system is rather sensitive to synthetic parameters including solvent, counteranions and temperature.

### 2.3. Permanent porosity   

**Figure 5:**
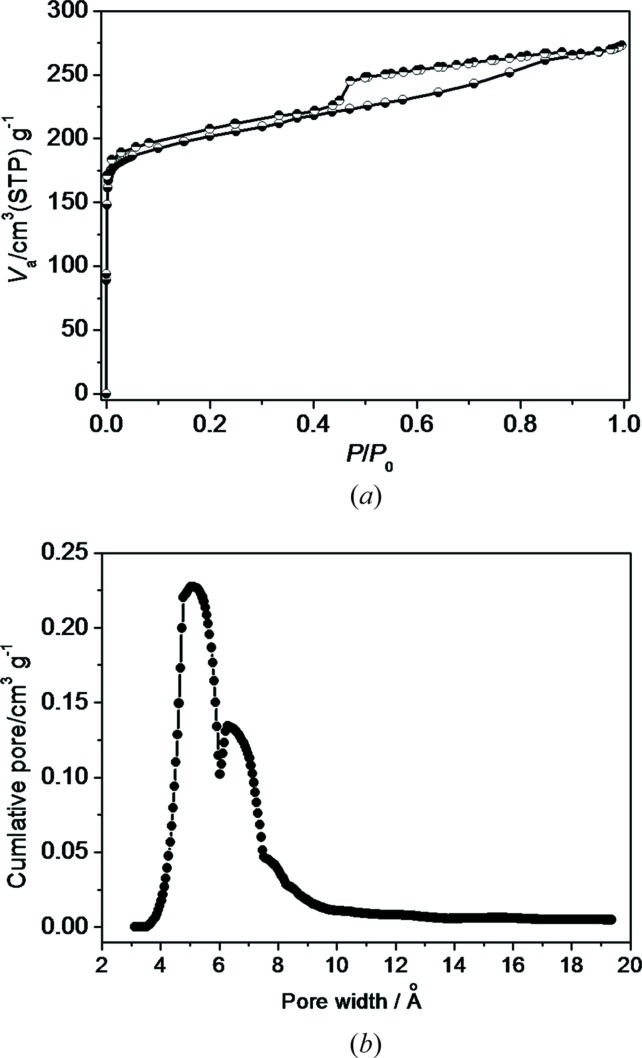
(*a*) N_2_ adsorption–desorption isotherms for T_imi_-Cu measured at 77 K and (*b*) Horvath–Kawazoe micropore size distribution.

To evaluate the permanent porosity, nitrogen physisorption measurements were performed at 77 K. Prior to analysis, pore activation was performed by evacuating T_imi_-Cu by thermal activation under vacuum at 358 K following surface cleaning by EtOH. This gives rise to a partially desolvated sample, in which the DMF molecules occluded within the channels were not removed completely (Seo *et al.*, 2010 ▸; Hijikata *et al.*, 2013 ▸; Wang *et al.*, 2013 ▸, 2015 ▸). FT–IR spectra confirm the presence of residual DMF (Fig. S7). An N_2_ adsorption isotherm of T_imi_-Cu reveals a steep uptake in the low-pressure region and the profile displays a type-I curve that is typical of microporous materials (Fig. 5 ▸). The Langmuir and BET surface areas are calculated to be 1145 and 771 m^2^ g^−1^, respectively, and the total pore volume is 0.31 cm^3^ g^−1^ (Table S3, Figs. S8–S10). Surprisingly T_imi_-Cu shows a double-peak pore size distribution centered around 5.1 and 6.8 Å according to the Horvath–Kawazoe method. The main pore size at 5.1 Å is consistent with other **rtl**-MOFs (Zhang *et al.*, 2010 ▸), while 6.8 Å is unprecedented (see the discussion below). For comparison, T_triaz_-Cu has Langmuir and BET surface areas of 893 and 768 m^2^ g^−1^, while those of T_tetraz_-Cu are 1055 and 766 m^2^ g^−1^, consistent with the reported data (Zhang *et al.*, 2010 ▸). The total pore volumes are 0.29 and 0.30 cm^3^ g^−1^, respectively, and the pore sizes are comparable at around 4.9 Å.

The pore enlargement of T_imi_-Cu is unexpected and can reasonably be related to the framework disorder. On one hand, the disorder in T_imi_-Cu causes the pore channels to become distorted; on the other hand, appearance of structural disorder in the crystal structure always implies concomitance of local defects due to coordination mismatch or topological distortion caused by interlayer gliding. Hence, enlargement of partial pores in T_imi_-Cu is understandable. The defects (probably containing uncoordinated metal centers) may be readily occupied by solvated DMF molecules. Moreover, the N_2_ sorption isotherms show an obvious H2-type hysteresis loop which is usually considered to be a characteristic of mesoporosity (Li *et al.*, 2013 ▸; Fang *et al.*, 2010 ▸; Zhao *et al.*, 2011 ▸; Qiu *et al.*, 2008 ▸), but we believe that this may also be attributed to the structural disorder. The clustering of numerous local defects may result in larger-scale mesoporosity, so the existence of some mesopores in the disordered framework is to be expected.

### 2.4. CO_2_ capture and framework dynamics   

**Figure 6:**
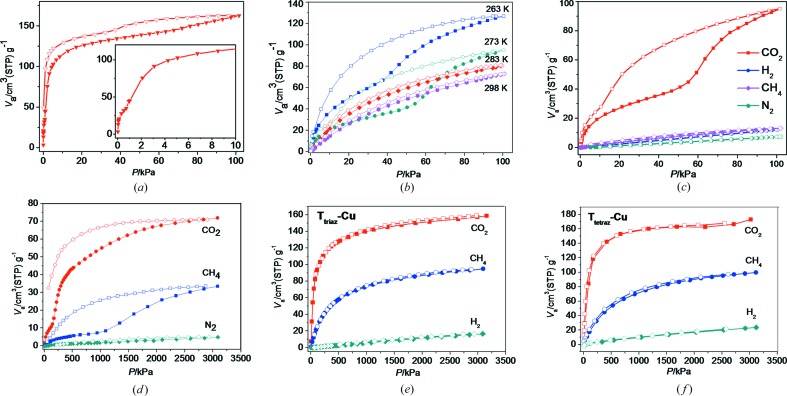
CO_2_ adsorption–desorption isotherms for T_imi_-Cu measured at (*a*) 195 K (insert shows an enlargement of the low-pressure section); (*b*) 263, 273, 283 and 298 K; and (*c*) CO_2_, N_2_, H_2_ and CH_4_ adsorption–desorption isotherms for T_imi_-Cu measured at 273 K, and CO_2_, N_2_ and CH_4_ adsorption–desorption isotherms for (*d*) T_imi_-Cu, (*e*) T_triaz_-Cu and (*f*) T_tetraz_-Cu measured under various pressures at 298 K.

Low-pressure and high-pressure CO_2_ sorption has been studied. As depicted in Fig. 6 ▸, T_imi_-Cu adsorbs significant amounts of CO_2_ at various temperatures (195, 263, 273, 283 and 298 K). At 195 K, the isotherms are type I, which is typical for microporous materials. T_imi_-Cu displays an uptake capacity of 7.3 mmol g^−1^ (32.0 wt%) for CO_2_ at 1 bar. A striking feature is that T_imi_-Cu also shows high CO_2_ uptake at 273 K. T_imi_-Cu has CO_2_ storage capacity of 4.2 mmol g^−1^ (18.7 wt%) at 273 K, 1 bar. The adsorption isotherm of CO_2_ up to 30 bar at 298 K indicates that T_imi_-Cu shows a CO_2_ uptake capacity of 3.2 mmol g^−1^ (14.1 wt%) at 30 bar. The high CO_2_ uptake capacity of T_imi_-Cu, which has no open metal/N-donor sites on the inner pore surface revealed by the X-ray structure, hints at other contributions, namely framework disorder (see the discussion below).

Another noteworthy feature is that T_imi_-Cu shows remarkable hysteretic sorption behavior toward CO_2_, while the isostructural T_triaz_-Cu and T_tetraz_-Cu do not show obvious hysteresis loops (Fig. 6 ▸). The stepwise sorption usually indicates filling of different types of pore sites, originating from the gate effect or dynamic nature (Kitaura *et al.*, 2002 ▸; Thallapally *et al.*, 2008 ▸; Chen, Ma, Hurtado *et al.*, 2007 ▸; Nouar *et al.*, 2012 ▸; Suzuki *et al.*, 2016 ▸; Carrington *et al.*, 2017 ▸; Taylor *et al.*, 2016 ▸; Choi & Suh, 2009 ▸; Llewellyn *et al.*, 2006 ▸) and framework defects (Yang *et al.*, 2012 ▸). Careful examination reveals that T_imi_-Cu exhibits stepwise adsorption under low pressure at 195 K (Fig. 6 ▸ *a*). Surprisingly, such adsorption/desorption hysteresis becomes more prominent at elevated temperatures up to 273 K (Fig. 6 ▸ *b*). Note that a higher pressure is needed to initiate the hysteresis as temperature increases. The inducing pressure of *P/P*_0_ = 0.01 at 195 K increases to 0.37 at 263 K and 0.54 at 273 K.

The adsorption isotherms of CO_2_ up to 30 bar at 298 K further reveal the temperature-gating pressure relationship (Fig. 6 ▸). T_imi_-Cu exhibits a distinct stepwise adsorption isotherm, while the desorption branch does not trace the adsorption branch, forming a remarkable hysteresis loop. At low pressure, only a small amount of CO_2_ (0.5 mmol g^−1^) is adsorbed. A sudden rise in the isotherm occurs at an inducing pressure of 125 kPa, which is higher than that at 273 K. This confirms the low-pressure observations that a higher pressure is needed to initiate the hysteresis as temperature increases.

Considering the above temperature-dependent variations of CO_2_ sorption capacity and hysteresis, and comparing the structural nature of these isoreticular **rtl**-MOFs, we believe that the framework disorder in T_imi_-Cu imparts an essential effect on the CO_2_ sorption behavior. As discussed above, the structural disorder causes the pore channels to become too distorted for gas molecules to permeate through, and renders some local defects which might be partly maintained by the solvated DMF molecules. If gas molecules have a tendency to interact with the pore surface (protruding heterocycles) and defect sites (such as uncoordinated metal centers), gas uptake may induce framework dynamics. For T_imi_, the blocking/shielding DMF molecules can facilitate and affect the structural transformation, thus making hysteresis pronounced. At low temperature, *i.e.* 195 K, the coordination framework and shielding DMF molecules are relatively static, and the hysteretic steps are therefore relatively inexplicit. As the temperature increases, the kinetically hindered pores in the disordered framework become easier to break through, thus displaying a larger hysteresis loop. However, once the temperature rises above 283 K, hysteresis turns indistinctive again (Fig. 6 ▸ *b*), implying facile and expeditious structural conversions above this temperature. Additionally, the inducing pressure of hysteresis increases with rise in temperature, which may be due to higher thermal vibration of the framework, hindering DMF and adsorbed CO_2_ molecules at elevated temperature and resulting in weaker adsorbent/adsorbate interactions, thus requiring a larger pressure to push the motion of the framework. This may also account for the observation that the CO_2_ uptake capacity of T_imi_-Cu decreases as temperature increases relative to that of T_triaz_-Cu and T_tetraz_-Cu. The CH_4_ isotherm also exhibits a broad hysteresis loop with an inflection point at 298 K and a higher pressure of *ca*. 940 kPa, which may relate to its larger polarizability (25.93 × 10^−25^ cm^3^) (Li *et al.*, 2009 ▸; Sircar, 2006 ▸). This offers a new strategy for hysteretic sorption of CH_4_ (Taylor *et al.*, 2016 ▸; Mason *et al.*, 2014 ▸).

According to the above discussion, the present CO_2_ uptake process may represent a distinct strategy to drive adsorption/desorption hysteresis that is inherently related to framework (pore) disorder. In contrast to the gate effect and interpenetrating dynamics (Kitaura *et al.*, 2002 ▸; Thallapally *et al.*, 2008 ▸; Chen, Ma, Hurtado *et al.*, 2007 ▸; Nouar *et al.*, 2012 ▸; Suzuki *et al.*, 2016 ▸; Carrington *et al.*, 2017 ▸; Taylor *et al.*, 2016 ▸; Choi & Suh, 2009 ▸; Llewellyn *et al.*, 2006 ▸), the present CO_2_ (and CH_4_) hysteresis is temperature dependent, originating from the propensity of structural disorder which can be affected by guest intrusion. The structure dynamics can be induced selectively by CO_2_ (and CH_4_ at higher pressure) but not by N_2_ or H_2_.

In order to have further insight into the structure dynamics, PXRD investigation was performed. PXRD patterns of T_imi_-Cu remain unchanged under a high-pressure CO_2_ atmosphere up to 3.0 MPa even if accompanied by pulverizing of the crystal sample (Fig. S11). Moreover, the XRD patterns of T_imi_-Cu show no change after tablet compression with a tablet press from 0 to 20 MPa (Fig. S12). The results reveal that the long-range order of T_imi_-Cu is generally maintained under high pressure. The XRD signals become weak above 30 MPa, showing partial loss of long-range order under high pressure. Therefore, either no phase transformation occurs or the structural change is tiny under high pressure.

Two possible factors may contribute to the CO_2_ hysteresis. (i) Penetration of CO_2_ and CH_4_ through the disordered pore channels offers a greater driving force than through the straight 1D channels, and interactions of CO_2_ with the protruding imidazole rings facilitate their rotation along N—C bonds. Simulation of the CO_2_ adsorption isotherms at 263 K shows that a slight rotation of the imidazole ring of 5.448° has a dramatic effect on the adsorption results (Fig. S13). (ii) DMF guest molecules in the framework perhaps block the pore entrance, which becomes dynamic when the temperature and/or pressure increases. Such framework dynamics may result in partial loss of long-range order, as shown by the broadened PXRD pattern after sorption. Therefore, the framework dynamics are responsive to CO_2_ uptake depending on feasible conditions created by proper temperature. This is analogous with the partially interpenetrated NOTT-202a which shows temperature-dependent adsorption/desorption hysteresis only below the triple point of CO_2_ (216.7 K), corresponding completely to the framework defects. In our case, the framework dynamics may be more related to the topological disorder, because the disordered structure model established by single-crystal analysis prefers topological distortion to defect formation. Nevertheless, interaction of CO_2_ with the defect sites should also contribute to the dynamics of the disordered framework.

The CO_2_ adsorption capacity around room temperature is essential for potential industrial usage, such as CO_2_ capture and separation in upgrading of natural gas (natural gas clean-up, CO_2_/CH_4_), post-combustion (flue gas, CO_2_/N_2_) and pre-combustion (shifted synthesis gas stream, CO_2_/H_2_) (Sumida *et al.*, 2012 ▸; Li, Sculley & Zhou, 2012 ▸; Nugent *et al.*, 2013 ▸; Bloch *et al.*, 2013 ▸). To investigate the sorption selectivity of T_imi_-Cu, CO_2_, N_2_, H_2_ and CH_4_ low-pressure adsorption isotherms were measured at 273 K, and CO_2_, N_2_ and CH_4_ high-pressure adsorption isotherms at 298 K, and compared in Figs. 6 ▸(*e*) and 6(*f*). For T_imi_-Cu, considerably larger amounts of CO_2_ are adsorbed than N_2_, H_2_ and CH_4_, suggesting that the gas uptake capacity drops remarkably for N_2_, H_2_ and CH_4_ but remains significant for CO_2_ at elevated temperatures (see Figs. S8–S10 for low-temperature data).

The above results verify that T_imi_-Cu has a high and general gas sorption selectivity for CO_2_ over H_2_/CH_4_/N_2_; the question is then how such sorption behavior happens. Various effects have been reported in the literature that enforce strong interactions between the host framework and CO_2_ under ambient conditions, *e.g.* immobilization of open metal sites or polarized functional groups (Yuan *et al.*, 2010 ▸; Sumida *et al.*, 2012 ▸; Cui *et al.*, 2012 ▸; Gu *et al.*, 2010 ▸; Demessence *et al.*, 2009 ▸; McDonald *et al.*, 2011 ▸; Banerjee *et al.*, 2009 ▸). In particular, aromatic ligands containing uncoordinated N donors (*e.g.* tetrazole-based ligands) were found to improve the selective adsorption behavior for CO_2_ (Zhang *et al.*, 2010 ▸; Cui *et al.*, 2012 ▸; Lin *et al.*, 2010 ▸, 2012 ▸; Qin *et al.*, 2012 ▸). In the present azole-based **rtl**-MOF, no purposely introduced open metal sites exist on the pore surface and T_imi_-Cu has no polarized functional groups (open N-donor sites). So, the good selectivity of T_imi_-Cu probably relies on the above-described structural disorder, as well as the tubular pore channels characteristic of **rtl**-MOFs containing pillaring T-shaped ligands.

First of all, the tubular channels of narrow size in the present **rtl**-MOF have proved important (Du *et al.*, 2013 ▸; An & Rosi, 2010 ▸). It is argued that, for MOFs with bigger pore sizes (>6 Å), substitution of C—H moieties with N donors does not significantly affect the adsorption capacity for H_2_ and CO_2_ (Park *et al.*, 2011 ▸). The pore sizes of T_imi_-Cu justify this assumption well. Second, the quadruple moment of CO_2_ renders a stronger interaction with the host framework, which contributes excess energy for CO_2_ to enter the pore channels. Since CO_2_ is well known to have stronger adsorbent/adsorbate interactions due to strong polarizability (29.11 × 10^−25^ cm^3^) and quadruple moment (4.30 × 10^−26^ esu cm^2^) (Li *et al.*, 2009 ▸; Sircar, 2006 ▸), selective hysteretic sorption of CO_2_ over N_2_, H_2_ and CH_4_ is understandable at low pressure. Third, the structural disorder may cause defects in the coordination framework, thus creating open metal sites. Such defects may exist in a small portion of the framework (see above). On the other hand, the framework disorder enables distorted pore channels as well as straight channels. The distorted channels with narrow size are appropriate for holding gas molecules kinetically within the channels, which increases the van der Waals interactions between the host framework and gas molecules (Wen *et al.*, 2012 ▸). Most importantly, as temperature rises, CO_2_ uptake towards such disordered pores amplifies the framework dynamics, resulting in selective sorption hysteresis. Such adsorption/desorption hysteretic behavior at room temperature is rare (Hijikata *et al.*, 2013 ▸; Wang *et al.*, 2013 ▸, 2015 ▸); it greatly improves the adsorption selectivity for CO_2_ at ambient temperature, similar to observations of selective CO_2_ capture by flexible or dynamic MOFs under different conditions (Choi & Suh, 2009 ▸; Llewellyn *et al.*, 2006 ▸; Mohamed *et al.*, 2012 ▸; Burd *et al.*, 2012 ▸; Eguchi *et al.*, 2012 ▸). In addition, the present framework disorder induces hysteretic sorption in a much broader range from 195 K to room temperature. This allows the capture of CO_2_ at high pressure, but leaves CO_2_ trapped in the pores at low pressure, thus facilitating the separation of CO_2_ from H_2_/CH_4_/N_2_ under more industrially applicable conditions.

## 3. Conclusions   

A unique **rtl**-MOF (T_imi_-Cu) with framework disorder was prepared by incorporating an imidazole ring into a T-shaped ligand and the gas sorption properties were evaluated. In contrast to other azole-based **rtl**-MOFs with five-membered-ring heterocycles (triazole, T_triaz_-Cu; tetrazole, T_tetraz_-Cu), T_imi_-Cu does not integrate open N-donor sites while containing only two C—H moieties in its characteristic tubular pores. Remarkably, T_imi_-Cu displays crystallographically identifiable disorder of the framework. Considering the interesting effects induced by structural disorder on framework materials (Cairns & Goodwin, 2013 ▸; Tucker *et al.*, 2005 ▸; Goodwin *et al.*, 2009 ▸; Cheetham *et al.*, 2016 ▸; Fang *et al.*, 2015 ▸; Tahier & Oliver, 2017 ▸; Cliffe *et al.*, 2014 ▸; Li *et al.*, 2013 ▸; Allan *et al.*, 2012 ▸; Amirjalayer & Schmid, 2008 ▸), T_imi_-Cu provides a unique example to investigate the effect of framework pore disorder on sorption properties. As a defective derivative, T_imi_-Cu retains the long-range order and topology of the parent framework of **rtl**-MOFs, while it exhibits pore disorder and a relatively large percentage of defects in an otherwise highly crystalline material. Single-crystal analyses establish the disordered structural model in relation to porosity, featuring distorted 1D tubular channels and DMF-guest-remediated defects. These factors endow T_imi_-Cu with good gas sorption capacity. Importantly, temperature-dependent hysteretic CO_2_ (and CH_4_) sorption is shown up to 298 K, which dramatically enhances selective adsorption of CO_2_ (and CH_4_) at elevated temperatures. Therefore, the present azole-based **rtl**-MOF shows strong binding with CO_2_ and high selectivity for CO_2_ over H_2_/CH_4_/N_2_ at ambient temperature. Furthermore, the results imply the significance of structure disorder (defects) on the modification of the performance of framework materials, providing a viewpoint for expanding the properties of framework materials.

## 4. Experimental   

### 4.1. Materials and methods   

All starting materials and solvents were obtained from commercial sources and used without further purification unless otherwise indicated. Di­methyl-5-(1*H*-imidazol-1-yl)isophthalate was prepared according to the published procedure (Wang *et al.*, 2013 ▸). PXRD data were recorded on a Bruker D8 Advance diffractometer at 40 kV and 40 mA with a Cu-target tube and a graphite monochromator. Infrared spectra were measured on a Nicolet/Nexus-670 FT–IR spectrometer with KBr pellets. Thermogravimetric analysis was performed under N_2_ at a heating rate of 10 K min^−1^ on a Netzsch Termo Microbalance TG 209 F3 Tarsus. The sorption isotherms were measured with a Quantachrome Autosorb-iQ or Autosorb-iQ2 analyzer.

### 4.2. Synthesis of 5-(1*H*-imidazol-1-yl)benzene-1,3-di­carboxylic acid (T_imi_)   

Ester hydrolysis of dimethyl-5-(1*H*-imidazol-1-yl)isophthalate was performed via an acid-catalyzed ester hydrolysis in HCl solution. Di­methyl-5-(1*H*-imidazol-1-yl)isophthalate (160 mg, 0.6 mmol) was refluxed for 36 h in 20% HCl (8 ml). The solvent was evaporated to obtain the product (140 mg, >99%). The product was soluble in DMF and MeOH. ^1^H NMR (300 MHz, DMSO-*d*_6_): δ 9.76 (*s*, 1H), 8.54 (*s*, 1H), 8.50 (*s*, 2H), 8.41 (*s*, 1H), 7.87 (*s*, 1H). IR (cm^−1^, KBr): 3163 (*w*), 3087 (*w*), 1711 (*m*), 1674 (*m*), 1600 (*w*), 1539 (*w*), 1399 (*m*), 1348 (*m*), 1232 (*s*), 1068 (*s*), 876 (*w*), 757 (*m*), 672 (*m*), 616 (*w*).

### 4.3. Synthesis of T_imi_-Cu   

A solution of T_imi_ (6.0 mg, 0.025 mmol) in DMF (3 ml) and a solution of CuCl_2_·2H_2_O (8.5 mg, 0.05 mmol) in EtOH (1 ml) were mixed. The resultant clear solution was heated in a closed vial at 353 K for 3 days. Green crystals were collected using filtration (7 mg, 70%). Microanalysis found (calculated) for C_11_H_6_O_4_N_2_Cu·1.5DMF·0.5H_2_O: C, 45.22 (45.15); H 4.28 (4.28); N 11.74 (11.89)%. FT–IR (cm^−1^, KBr): 3426 (*b*), 3101 (*w*), 2929 (*w*), 1634 (*m*), 1594 (*m*), 1503 (*w*), 1385 (*s*), 1249 (*w*), 1070 (*m*), 922 (*w*), 782 (*w*), 730 (*m*), 656 (*w*). For thermally activated product under vacuum at 358 K following CH_2_Cl_2_ solvent exchange, microanalysis found (calculated) for C_11_H_6_O_4_N_2_Cu·3H_2_O: C 37.34 (37.99), H 3.55 (3.48), N 7.82 (8.06)%.

### 4.4. X-ray structure determination   

X-ray reflection data were collected at 150 (2) K on an Oxford Gemini S Ultra diffractometer equipped with a graphite-monochromated Enhance (Cu) X-ray source (λ = 1.54178 Å). An empirical absorption correction was applied to the intensity data using spherical harmonics implemented in the *SCALE3 ABSPACK* scaling algorithm (Agilent, 2012 ▸). The structure was solved by direct methods following difference Fourier syntheses and was refined by the full matrix least-squares method against *F*_o_^2^ using *SHELXTL* software (Sheldrick, 2015 ▸). The whole framework is disordered over two positions with an occupancy ratio of 0.694:0.306. The unit-cell volume includes a large region of disordered solvent (1.5 DMF and 0.5 H_2_O molecules). One DMF molecule is disordered over two positions with an occupancy ratio of 0.679:0.321. There are 0.25 water and 0.25 DMF molecules located at the inversion center. Modeled refinements were applied to the disordered parts including the imidazole ring, solvated water and DMF molecules to make them geometrically reasonable, resulting in a total of 1074 restraints.

Crystallographic data for T_imi_-Cu: C_15.5_H_17_CuN_3.5_O_5.75_, FW = 407.87, monoclinic, *P*2_1_/*c*, *a* = 10.8431 (6) Å, *b* = 11.8835 (6) Å, *c* = 14.4823 (9) Å, α = 90°, β = 109.361 (7)°, γ = 90°, *V* = 1760.57 (17) Å^3^, *Z* = 4, *T* = 150 (2) K, λ = 1.54178 Å, ρ_calc_ = 1.539 mg m^−3^, μ = 2.097 mm^−1^, 4661 reflections were collected (2553 were unique) for 4.93 < θ < 59.98, *R*(int) = 0.0325, *R*_1_ = 0.0840, *wR*_2_ = 0.2296 [*I* > 2σ(*I*)], *R*_1_ = 0.0984, *wR*_2_ = 0.2447 (all data) for 258 parameters, GOF = 1.073, CCDC reference 945810.

## 5. Related literature   

The following references are cited in the supporting information: Vitillo *et al.* (2008 ▸); Zhou *et al.* (2008 ▸).

## Supplementary Material

Crystal structure: contains datablock(s) I. DOI: 10.1107/S2052252518015749/lq5014sup1.cif

Supporting information file. DOI: 10.1107/S2052252518015749/lq5014sup2.pdf

CCDC reference: 945810

## References

[bb1] Agilent, (2012). *CrysAlis PRO.* Agilent Technologies, Oxford, England.

[bb2] Alhamami, M., Doan, H. & Cheng, C H. (2014). *Materials*, **7**, 3198–3250. 10.3390/ma7043198 PMC5453333 28788614

[bb3] Allan, P. K., Chapman, K. W., Chupas, P. J., Hriljac, J. A., Renouf, C. L., Lucas, T. C. A. & Morris, R. E. (2012). *Chem. Sci.* **3**, 2559–2564.

[bb4] Amirjalayer, S. & Schmid, R. (2008). *J. Phys. Chem. C*, **112**, 14980–14987.

[bb5] An, J. & Rosi, N. L. (2010). *J. Am. Chem. Soc.* **132**, 5578–5579. 10.1021/ja1012992 20373762

[bb6] Bae, Y.-S., Lee, C. Y., Kim, K. C., Farha, O. K., Nickias, P., Hupp, J. T., Nguyen, S. T. & Snurr, R. Q. (2012). *Angew. Chem. Int. Ed.* **51**, 1857–1860. 10.1002/anie.201107534 22250050

[bb7] Banerjee, R., Furukawa, H., Britt, D., Knobler, C., O’Keeffe, M. & Yaghi, O. M. (2009). *J. Am. Chem. Soc.* **131**, 3875–3877. 10.1021/ja809459e 19292488

[bb8] Bloch, W. M., Babarao, R., Hill, M. R., Doonan, C. J. & Sumby, C. J. (2013). *J. Am. Chem. Soc.* **135**, 10441–10448. 10.1021/ja4032049 23758473

[bb9] Bourne, S. A., Lu, J., Mondal, A., Moulton, B. & Zaworotko, M. J. (2001). *Angew. Chem. Int. Ed.* **40**, 2111–2113. 11433460

[bb10] Burd, S. D., Ma, S., Perman, J. A., Sikora, B. J., Snurr, R. Q., Thallapally, P. K., Tian, J., Wojtas, L. & Zaworotko, M. J. (2012). *J. Am. Chem. Soc.* **134**, 3663–3666. 10.1021/ja211340t 22316279

[bb11] Cairns, A. B. & Goodwin, A. L. (2013). *Chem. Soc. Rev.* **42**, 4881–4893. 10.1039/c3cs35524a 23471316

[bb12] Carrington, E. J., McAnally, C. A., Fletcher, A. J., Thompson, S. P., Warren, M. & Brammer, L. (2017). *Nat. Chem.* **9**, 882–889. 10.1038/nchem.2747 28837170

[bb13] Caskey, S. R., Wong-Foy, A. G. & Matzger, A. J. (2008). *J. Am. Chem. Soc.* **130**, 10870–10871. 10.1021/ja8036096 18661979

[bb14] Cheetham, A. K., Bennett, T. D., Coudert, F. X. & Goodwin, A. L. (2016). *Dalton Trans.* **45**, 4113–4126. 10.1039/c5dt04392a 26836459

[bb17] Chen, B., Fronczek, F. R., Courtney, B. H. & Zapata, F. (2006). *Cryst. Growth Des.* **6**, 825–828.

[bb18] Chen, B., Ma, S., Hurtado, E. J., Lobkovsky, E. B. & Zhou, H.-C. (2007). *Inorg. Chem.* **46**, 8490–8492. 10.1021/ic7014034 17854181

[bb19] Chen, B., Ma, S., Zapata, F., Fronczek, F. R., Lobkovsky, E. B. & Zhou, H. C. (2007). *Inorg. Chem.* **46**, 1233–1236. 10.1021/ic0616434 17291116

[bb20] Chen, B., Ma, S., Zapata, F., Lobkovsky, E. B. & Yang, J. (2006). *Inorg. Chem.* **45**, 5718–5720. 10.1021/ic060437t 16841969

[bb16] Chen, M.-S., Chen, M., Okamura, T., Sun, W. & Ueyama, N. (2011). *Microporous Mesoporous Mater.* **139**, 25–30.

[bb15] Chen, Z., Adil, K., Weseliński, J., Belmabkhout, Y. & Eddaoudi, M. (2015). *J. Mater. Chem. A*, **3**, 6276–6281.

[bb21] Cheng, F., Li, Q., Duan, J., Hosono, N., Noro, S., Krishna, R., Lyu, H., Kusaka, S., Jin, W. & Kitagawa, S. (2017). *J. Mater. Chem. A*, **5**, 17874–17880.

[bb22] Choi, H.-S. & Suh, M. P. (2009). *Angew. Chem. Int. Ed.* **48**, 6865–6869. 10.1002/anie.200902836 19693760

[bb23] Cliffe, M. J., Wan, W., Zou, X., Chater, P. A., Kleppe, A. K., Tucker, M. G., Wilhelm, H., Funnell, N. P., Coudert, F. X. & Goodwin, A. L. (2014). *Nat. Commun.* **5**, 4176. 10.1038/ncomms5176 PMC4730551 24946837

[bb24] Colombo, V., Montoro, C., Maspero, A., Palmisano, G., Masciocchi, N., Galli, S., Barea, E. & Navarro, J. A. R. (2012). *J. Am. Chem. Soc.* **134**, 12830–12843. 10.1021/ja305267m 22765315

[bb25] Cui, P., Ma, Y. G., Li, H. H., Zhao, B., Li, J. R., Cheng, P., Balbuena, P. B. & Zhou, H. C. (2012). *J. Am. Chem. Soc.* **134**, 18892–18895. 10.1021/ja3063138 23113600

[bb26] Das, M. C., Guo, Q., He, Y., Kim, J., Zhao, C. G., Hong, K., Xiang, S., Zhang, Z., Thomas, K. M., Krishna, R. & Chen, B. (2012). *J. Am. Chem. Soc.* **134**, 8703–8710. 10.1021/ja302380x 22545712

[bb27] Demessence, A., D’Alessandro, D. M., Foo, M. L. & Long, J. R. (2009). *J. Am. Chem. Soc.* **131**, 8784–8786. 10.1021/ja903411w 19505094

[bb28] Deng, H., Grunder, S., Cordova, K. E., Valente, C., Furukawa, H., Hmadeh, M., Gándara, F., Whalley, A. C., Liu, Z., Asahina, S., Kazumori, H., O’Keeffe, M., Terasaki, O., Stoddart, J. F. & Yaghi, O. M. (2012). *Science*, **336**, 1018–1023. 10.1126/science.1220131 22628651

[bb29] Du, L., Lu, Z., Zheng, K., Wang, J., Zheng, X., Pan, Y., You, X. & Bai, J. (2013). *J. Am. Chem. Soc.* **135**, 562–565. 10.1021/ja309992a 23268731

[bb30] Dybtsev, D. N., Chun, H. & Kim, K. (2004). *Angew. Chem. Int. Ed.* **43**, 5033–5036. 10.1002/anie.200460712 15384114

[bb31] Eddaoudi, M., Kim, J., Rosi, N., Vodak, D., Wachter, J., O’Keeffe, M. & Yaghi, O. M. (2002). *Science*, **295**, 469–472. 10.1126/science.1067208 11799235

[bb32] Eguchi, R., Uchida, S. & Mizuno, N. (2012). *Angew. Chem. Int. Ed.* **51**, 1635–1639. 10.1002/anie.201107906 22311812

[bb33] Eubank, J. F., Wojtas, L., Hight, M. R., Bousquet, T., Kravtsov, V. Ch. & Eddaoudi, M. (2011). *J. Am. Chem. Soc.* **133**, 17532–17535. 10.1021/ja203898s 21675767

[bb35] Fang, Q. R., Makal, T. A., Young, M. D. & Zhou, H. C. (2010). *Comments Inorg. Chem.* **31**, 165–195.

[bb34] Fang, Z., Bueken, B., De Vos, D. E. & Fischer, R. A. (2015). *Angew. Chem. Int. Ed.* **54**, 7234–7254. 10.1002/anie.201411540 PMC4510710 26036179

[bb36] Gao, L., Zhao, B., Li, G., Shi, Z. & Feng, S. (2003). *Inorg. Chem. Commun.* **6**, 1249–1251.

[bb37] Goodwin, A. L., Dove, M. T., Chippindale, A. M., Hibble, S. J., Pohl, A. H. & Hannon, A. C. (2009). *Phys. Rev. B*, **80**, 054101.

[bb38] Gu, J.-M., Kwon, T.-H., Park, J.-H. & Huh, S. (2010). *Dalton Trans.* **39**, 5608–5610. 10.1039/c0dt00392a 20498861

[bb39] Hijikata, Y., Horike, S., Sugimoto, M., Inukai, M., Fukushima, T. & Kitagawa, S. (2013). *Inorg. Chem.* **52**, 3634–3642. 10.1021/ic302006x 23496290

[bb40] Horike, S., Shimomura, S. & Kitagawa, S. (2009). *Nat. Chem.* **1**, 695–704. 10.1038/nchem.444 21124356

[bb41] Jia, J., Athwal, H. S., Blake, A. J., Champness, N. R., Hubberstey, P. & Schröder, M. (2011). *Dalton Trans.* **40**, 12342–12349. 10.1039/c1dt10901d 22024757

[bb42] Kitaura, R., Fujimoto, K., Noro, S., Kondo, M. & Kitagawa, S. (2002). *Angew. Chem. Int. Ed.* **41**, 133–135. 10.1002/1521-3773(20020104)41:1<133::aid-anie133>3.0.co;2-r 12491462

[bb43] Kobalz, M., Lincke, J., Kobalz, K., Erhart, O., Bergmann, J., Lässig, D., Lange, M., Möllmer, J., Gläser, R., Staudt, R. & Krautscheid, H. (2016). *Inorg. Chem.* **55**, 3030–3039. 10.1021/acs.inorgchem.5b02921 26950305

[bb47] Li, B., Zhang, Z., Li, Y., Yao, K., Zhu, Y., Deng, Z., Yang, F., Zhou, X., Li, G., Wu, H., Nijem, N., Chabal, Y. J., Lai, Z., Han, Y., Shi, Z., Feng, S. & Li, J. (2012). *Angew. Chem. Int. Ed.* **51**, 1412–1415. 10.1002/anie.201105966 22213672

[bb44] Li, J.-R., Kuppler, R. J. & Zhou, H.-C. (2009). *Chem. Soc. Rev.* **38**, 1477–1504. 10.1039/b802426j 19384449

[bb45] Li, J.-R., Sculley, J. & Zhou, H.-C. (2012). *Chem. Rev.* **112**, 869–932. 10.1021/cr200190s 21978134

[bb46] Li, L., Xiang, S., Cao, S., Zhang, J., Ouyang, G., Chen, L. & Su, C. Y. (2013). *Nat. Commun.* **4**, 1774. 10.1038/ncomms2757 PMC3644084 23653186

[bb49] Lin, J.-B., Zhang, J.-P. & Chen, X.-M. (2010). *J. Am. Chem. Soc.* **132**, 6654–6656. 10.1021/ja1009635 20420376

[bb48] Lin, Q., Wu, T., Zheng, S.-T., Bu, X. & Feng, P. (2012). *J. Am. Chem. Soc.* **134**, 784–787. 10.1021/ja2092882 22242771

[bb50] Llewellyn, P. L., Bourrelly, S., Serre, C., Filinchuk, Y. & Férey, G. (2006). *Angew. Chem. Int. Ed.* **45**, 7751–7754. 10.1002/anie.200602278 17054296

[bb51] Lü, X. Q., Qiao, Y., He, J., Pan, M., Kang, B. & Su, C. (2006). *Cryst. Growth Des.* **6**, 1910–1914.

[bb52] Ma, L., Falkowski, J. M., Abney, C. & Lin, W. (2010). *Nat. Chem.* **2**, 838–846. 10.1038/nchem.738 20861899

[bb53] Makal, T. A., Yakovenko, A. A. & Zhou, H.-C. (2011). *J. Phys. Chem. Lett.* **2**, 1682–1689. 10.1021/jz200424h 39506258

[bb54] Mason, J. A., Veenstra, M. & Long, J. R. (2014). *Chem. Sci.* **5**, 32–51.

[bb55] McDonald, T. M., D’Alessandro, D. M., Krishna, R. & Long, J. R. (2011). *Chem. Sci.* **2**, 2022–2028.

[bb56] McDonald, T. M., Lee, W. R., Mason, J. A., Wiers, B. M., Hong, C. S. & Long, J. R. (2012). *J. Am. Chem. Soc.* **134**, 7056–7065. 10.1021/ja300034j 22475173

[bb57] Mohamed, M. H., Elsaidi, S. K., Wojtas, L., Pham, T., Forrest, K. A., Tudor, B., Space, B. & Zaworotko, M. J. (2012). *J. Am. Chem. Soc.* **134**, 19556–19559. 10.1021/ja309452y 23170983

[bb58] Nouar, F., Devic, T., Chevreau, H., Guillou, N., Gibson, E., Clet, G., Daturi, M., Vimont, A., Grenèche, J. M., Breeze, M. I., Walton, R. I., Llewellyn, P. L. & Serre, C. (2012). *Chem. Commun.* **48**, 10237–10239. 10.1039/c2cc35348b 22968060

[bb59] Nugent, P., Belmabkhout, Y., Burd, S. D., Cairns, A. J., Luebke, R., Forrest, K., Pham, T., Ma, S., Space, B., Wojtas, L., Eddaoudi, M. & Zaworotko, M. J. (2013). *Nature*, **495**, 80–84. 10.1038/nature11893 23446349

[bb61] Park, J., Wang, Z. U., Sun, L.-B., Chen, Y.-P. & Zhou, H.-C. (2012). *J. Am. Chem. Soc.* **134**, 20110–20116. 10.1021/ja3085884 23157426

[bb60] Park, T.-H., Cychosz, K. A., Wong-Foy, A. G., Dailly, A. & Matzger, A. J. (2011). *Chem. Commun.* **47**, 1452–1454. 10.1039/c0cc03482g 21132184

[bb62] Pichon, A., Fierro, C. M., Nieuwenhuyzen, M. & James, S. L. (2007). *CrystEngComm*, **9**, 449–451.

[bb63] Qin, J.-S., Du, D., Li, W., Zhang, J., Li, S., Su, Z., Wang, X., Xu, Q., Shao, K. & Lan, Y. (2012). *Chem. Sci.* **3**, 2114–2118.

[bb64] Qiu, L. G., Xu, T., Li, Z., Wang, W., Wu, Y., Jiang, X., Tian, X. & Zhang, L. (2008). *Angew. Chem. Int. Ed.* **47**, 9487–9491. 10.1002/anie.200803640 18972472

[bb65] Rodríguez-Albelo, L. M., López-Maya, E., Hamad, S., Ruiz-Salvador, A. R., Calero, S. & Navarro, J. A. R. (2017). *Nat. Commun.* **8**, 14457. 10.1038/ncomms14457 PMC5316851 28198376

[bb66] Schneemann, A., Bon, V., Schwedler, I., Senkovska, I., Kaskel, S. & Fischer, R. A. (2014). *Chem. Soc. Rev.* **43**, 6062–6096. 10.1039/c4cs00101j 24875583

[bb67] Seki, K. & Mori, W. (2002). *J. Phys. Chem. B*, **106**, 1380–1385.

[bb68] Seo, J., Jin, N. & Chun, H. (2010). *Inorg. Chem.* **49**, 10833–10839. 10.1021/ic1012523 21062027

[bb69] Sheldrick, G. M. (2015). *Acta Cryst.* C**71**, 3–8.

[bb70] Sircar, S. (2006). *Ind. Eng. Chem. Res.* **45**, 5435–5448.

[bb71] Suh, M. P., Park, H. J., Prasad, T. K. & Lim, D.-W. (2012). *Chem. Rev.* **112**, 782–835. 10.1021/cr200274s 22191516

[bb72] Sumida, K., Rogow, D. L., Mason, J. A., McDonald, T. M., Bloch, E. D., Herm, Z. R., Bae, T. H. & Long, J. R. (2012). *Chem. Rev.* **112**, 724–781. 10.1021/cr2003272 22204561

[bb73] Sumida, K., Stück, D., Mino, L., Chai, J. D., Bloch, E. D., Zavorotynska, O., Murray, L. J., Dincă, M., Chavan, S., Bordiga, S., Head-Gordon, M. & Long, J. R. (2013). *J. Am. Chem. Soc.* **135**, 1083–1091. 10.1021/ja310173e 23244036

[bb74] Suzuki, T., Kotani, R., Kondo, A. & Maeda, K. (2016). *J. Phys. Chem. C*, **120**, 21571–21579.

[bb75] Tahier, T. & Oliver, C. L. (2017). *CrystEngComm*, **19**, 3607–3618.

[bb76] Taylor, J. M., Dekura, S., Ikeda, R. & Kitagawa, H. (2015). *Chem. Mater.* **27**, 2286–2289.

[bb77] Taylor, J. M., Komatsu, T., Dekura, S., Otsubo, K., Takata, M. & Kitagawa, H. (2015). *J. Am. Chem. Soc.* **137**, 11498–11506. 10.1021/jacs.5b07267 26302312

[bb78] Taylor, M. K., Runčevski, T., Oktawiec, J., Gonzalez, M. I., Siegelman, R. L., Mason, J. A., Ye, J., Brown, C. M. & Long, J. R. (2016). *J. Am. Chem. Soc.* **138**, 15019–15026. 10.1021/jacs.6b09155 27804295

[bb79] Thallapally, P. K., Tian, J., Radha Kishan, M., Fernandez, C. A., Dalgarno, S. J., McGrail, P. B., Warren, J. E. & Atwood, J. L. (2008). *J. Am. Chem. Soc.* **130**, 16842–16843. 10.1021/ja806391k 19053477

[bb80] Tranchemontagne, D. J., Mendoza-Cortés, J. L., O’Keeffe, M. & Yaghi, O. M. (2009). *Chem. Soc. Rev.* **38**, 1257–1283. 10.1039/b817735j 19384437

[bb81] Trickett, C. A., Gagnon, K. J., Lee, S., Gándara, F., Bürgi, H. & Yaghi, O. M. (2015). *Angew. Chem. Int. Ed.* **54**, 11162–11167. 10.1002/anie.201505461 26352027

[bb82] Tucker, M. G., Keen, D. A., Dove, M. T. & Trachenko, K. (2005). *J. Phys. Condens. Matter*, **17**, S67–S75.

[bb201] Vitillo, J. G. *et al.* (2008) *J. Am. Chem. Soc.* **130**, 8386–8396. 10.1021/ja8007159 18533719

[bb83] Wang, C., Li, L., Bell, J. G., Lv, X., Tang, S., Zhao, X. & Thomas, K. M. (2015). *Chem. Mater.* **27**, 1502–1516.

[bb84] Wang, S., Yang, Q., Zhang, J., Zhang, X., Zhao, C., Jiang, L. & Su, C. Y. (2013). *Inorg. Chem.* **52**, 4198–4204. 10.1021/ic301781n 23531233

[bb85] Wei, Y. S., Lin, R., Wang, P., Liao, P., He, C., Xue, W., Hou, L., Zhang, W., Zhang, J. & Chen, X. (2014). *CrystEngComm*, **16**, 6325–6330.

[bb86] Wen, L., Shi, W., Chen, X., Li, H. & Cheng, P. (2012). *Eur. J. Inorg. Chem.* **2012**, 3562–3568.

[bb87] Wilmer, C. E., Leaf, M., Lee, C. Y., Farha, O. K., Hauser, B. G., Hupp, J. T. & Snurr, R. Q. (2012). *Nat. Chem.* **4**, 83–89. 10.1038/nchem.1192 22270624

[bb88] Xiang, S., Huang, J., Li, L., Zhang, J., Jiang, L., Kuang, X. & Su, C. Y. (2011). *Inorg. Chem.* **50**, 1743–1748. 10.1021/ic102188v 21247087

[bb89] Xue, D.-X., Lin, Y.-Y., Cheng, X.-N. & Chen, X.-M. (2007). *Cryst. Growth Des.* **7**, 1332–1336.

[bb90] Yang, S., Lin, X., Lewis, W., Suyetin, M., Bichoutskaia, E., Parker, J. E., Tang, C. C., Allan, D. R., Rizkallah, P. J., Hubberstey, P., Champness, N. R., Mark Thomas, K., Blake, A. J. & Schröder, M. (2012). *Nat. Mater.* **11**, 710–716. 10.1038/nmat3343 22660661

[bb91] Yang, S., Liu, L., Sun, J., Thomas, K. M., Davies, A. J., George, M. W., Blake, A. J., Hill, A. H., Fitch, A. N., Tang, C. C. & Schröder, M. (2013). *J. Am. Chem. Soc.* **135**, 4954–4957. 10.1021/ja401061m 23485063

[bb93] Yuan, D., Zhao, D., Sun, D. & Zhou, H.-C. (2010). *Angew. Chem. Int. Ed.* **49**, 5357–5361. 10.1002/anie.201001009 20544763

[bb92] Yuan, S., Qin, J. S., Zou, L., Chen, Y. P., Wang, X., Zhang, Q. & Zhou, H. C. (2016). *J. Am. Chem. Soc.* **138**, 6636–6642. 10.1021/jacs.6b03263 27151517

[bb96] Zhang, J. P., Zhou, H. L., Zhou, D. D., Liao, P. Q. & Chen, X. M. (2017). *Natl. Sci. Rev.*, nwx127.

[bb94] Zhang, S.-M., Chang, Z., Hu, T.-L. & Bu, X.-H. (2010). *Inorg. Chem.* **49**, 11581–11586. 10.1021/ic1017467 21105646

[bb95] Zhang, Z., Gao, W., Wojtas, L., Ma, S., Eddaoudi, M. & Zaworotko, M. J. (2012). *Angew. Chem. Int. Ed.* **51**, 9330–9334. 10.1002/anie.201203594 22907717

[bb97] Zhao, Y., Zhang, J., Han, B., Song, J., Li, J. & Wang, Q. (2011). *Angew. Chem. Int. Ed.* **50**, 636–639. 10.1002/anie.201005314 21226141

[bb98] Zhong, R.-Q., Zou, R.-Q. & Xu, Q. (2011). *CrystEngComm*, **13**, 577–584.

[bb200] Zhou, W., Wu, H. & Yildirim, T. (2008) *J. Am. Chem. Soc.* **130**, 15268–15269. 10.1021/ja807023q 18950163

[bb99] Zhu, S. L., Ou, S., Zhao, M., Shen, H. & Wu, C.-D. (2015). *Dalton Trans.* **44**, 2038–2041. 10.1039/c4dt03371j 25515613

[bb100] Zou, R.-Q., Sakurai, H., Han, S., Zhong, R.-Q. & Xu, Q. (2007). *J. Am. Chem. Soc.* **129**, 8402–8403. 10.1021/ja071662s 17579408

