# Supplementary material for: Framework disorder and its effect on selective hysteretic sorption of a T-shaped azole-based metal–organic framework
Source: IUCrJ. 2019 Jan 1;6(Pt 1):85–95. doi: 10.1107/S2052252518015749 (PMC6327186; doi:10.1107/S2052252518015749)
Supplement: Supplementary file 2 [file m-06-00085-sup2.pdf]

IUCrJ

Volume 6 (2019)

Supporting information for article:

Framework disorder and effect on selective hysteretic sorption of T-shape-azole-based metal-organic framework

Sujuan Wang, Zhang-Wen Wei, Jianyong Zhang, Long Jiang, Dingxin Liu, Ji-Jun Jiang, Rui Si and Cheng-Yong Su

S1. Structure characterization

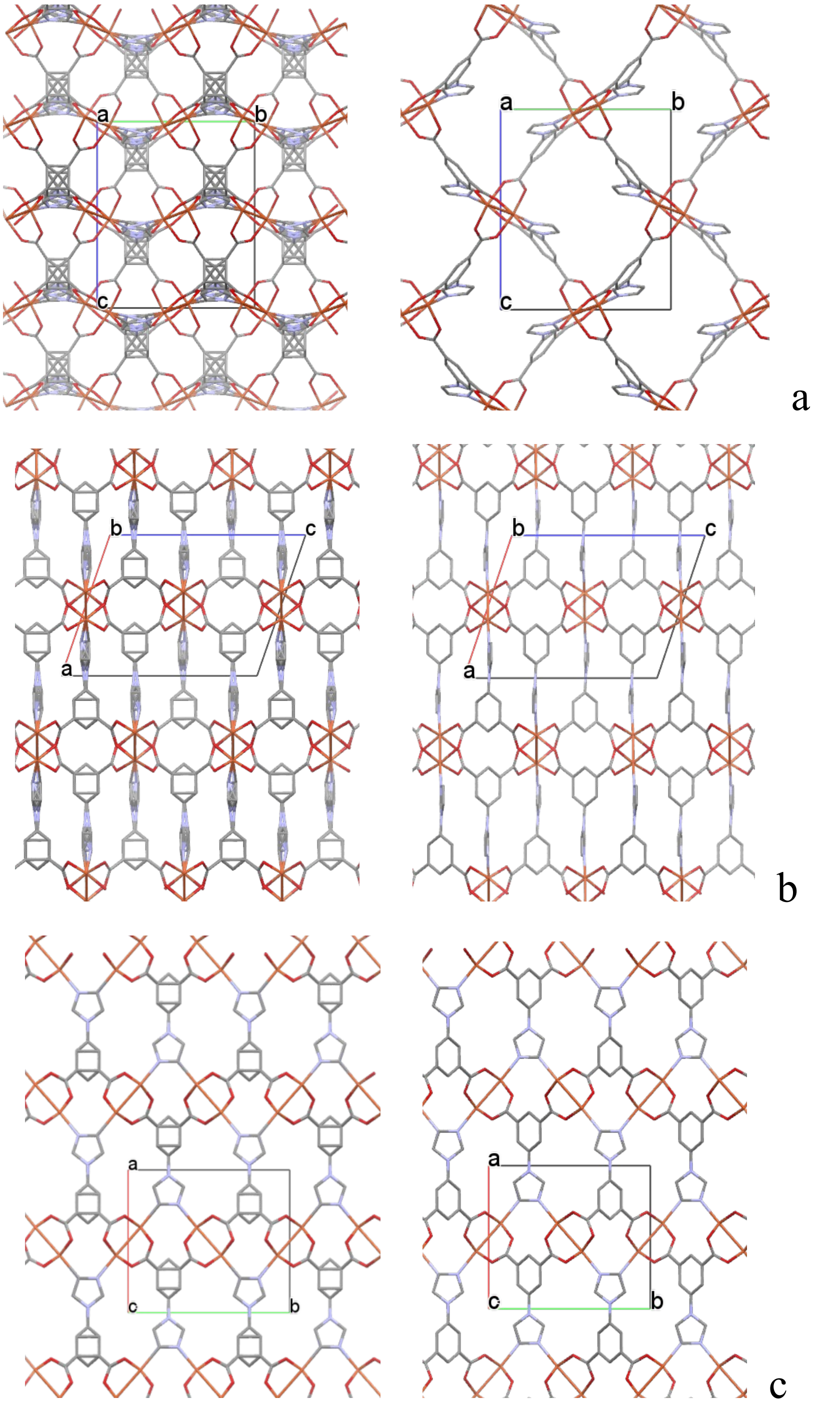

Figure S1 Comparison of the lattice packing of the disordered (left) and notionally ordered (right) framework of **T**$_{\mathbf{imi}}$**-Cu** in *a* (a), *b* (b) and *c* (c) directions. Solvated molecules and H atoms are omitted for clarity.

Table S1 Comparison of crystal data of azole-based rtl-MOFs, **T$_{imi}$-Cu**, **T$_{triaz}$-Cu** and **T$_{tetraz}$-Cu**.

	**T$_{imi}$-Cu** (this work) disordered	**T$_{imi}$-Cu** (NTU-11)[1]	**T$_{triaz}$-Cu**[2]	**T$_{tetraz}$-Cu**[3]
Empirical formula	$C_{15.5}H_{17}CuN_{3.5}O_{5.75}$	$C_{11}H_6CuN_2O_4$	$C_{10}H_5CuN_3O_4$	$C_{12}H_{13}CuN_5O_6$
Formula weight	407.87	293.72	294.71	386.81
T/K	150.0(1)	293(2)	100(2)	113(2)
Wavelength/Å	1.5418	0.71073	0.71073	0.71073
Crystal system	Monoclinic	Monoclinic	Monoclinic	Monoclinic
Space group	*P*2(1)/*c*	*P*2(1)/*c*	*P*2(1)/*c*	*P*2(1)/*c*
Unit cell dimensions				
a/Å	10.8431(6)	10.830(7)	10.884(5)	10.922(2)
b/Å	11.8835(6)	11.889(8)	12.049(6)	11.402(2)
c/Å	14.4823(9)	14.559(9)	14.338(7)	14.654(3)
α/°	90	90	90	90
β/°	109.361(7)	109.594(8)	109.552(7)	109.30(3)
γ/°	90	90	90	90
V/ Å^3	1760.57(17)	1766(2)	1772.0(15)	1722.3(6)
Z	4	4	4	4

1. Cheng, F. *et al.* (2017) *J. Mater. Chem. A* **5**, 17874-17880.

2. Eubank, J. F. *et al.* (2011) *J. Am. Chem. Soc.* **133**, 17532-17535.

3. Zhang, S.-M., Chang, Z., Hu, T.-L. & Bu, X.-H. (2010) *Inorg. Chem.* **49**, 11581-11586.

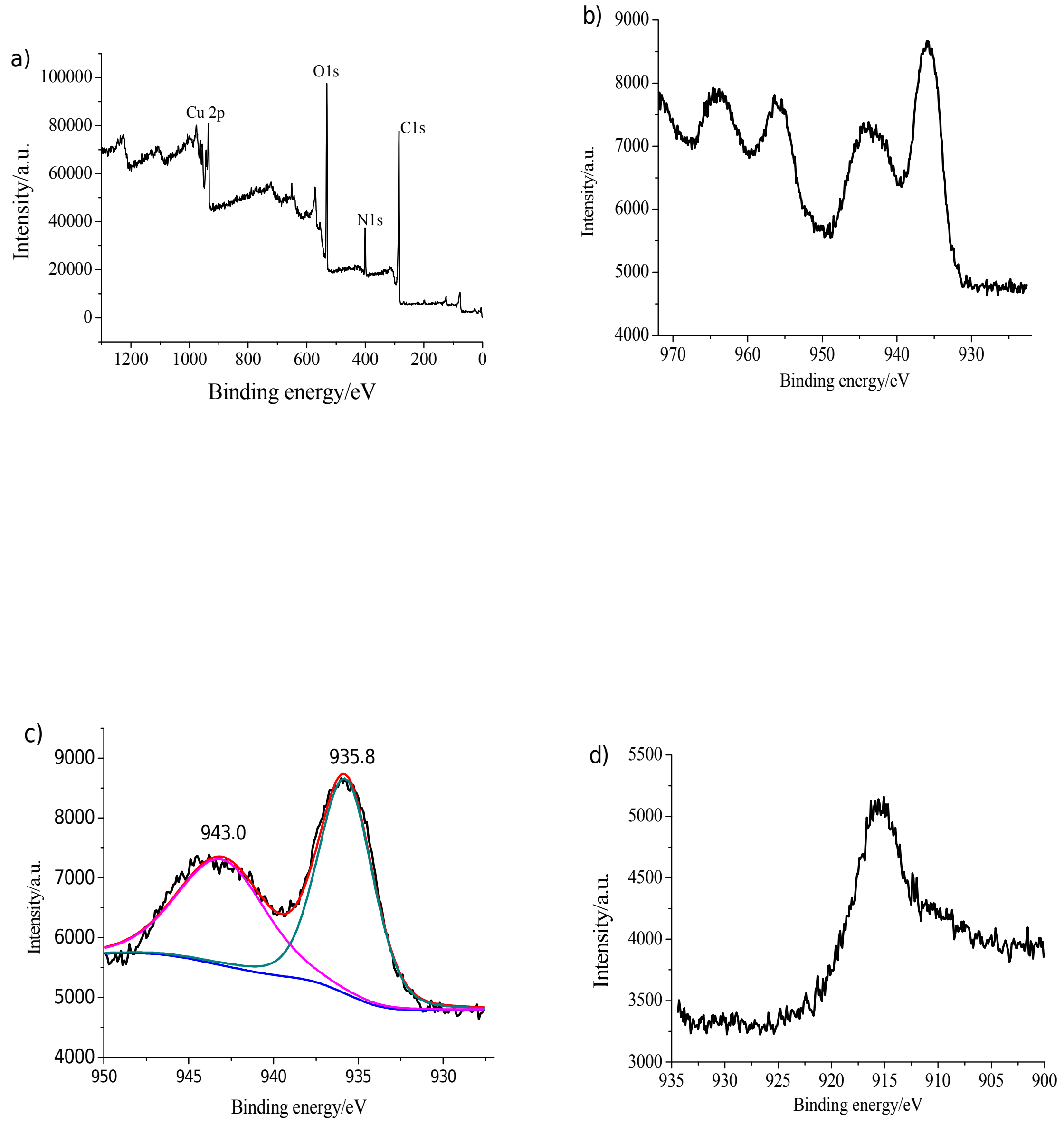

Figure S2 XPS spectra of **T_{imi}-Cu**, a) survey, b) Cu 2p, c) Cu $2p_{3/2}$, and d) Cu LMM spectra.

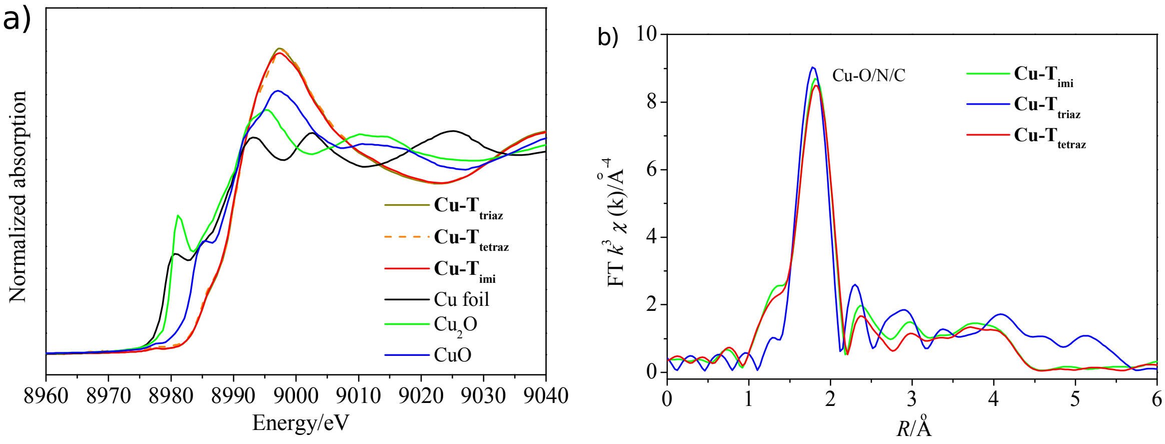

Figure S3 a) Cu K-edge XANES spectra for **Cu-T**$_{imi}$, **Cu-T**$_{triaz}$, **Cu-T**$_{tetraz}$, Cu foil, Cu_2O and CuO. b) Cu K-edge EXAFS in *R* space for **Cu-T**$_{imi}$, **Cu-T**$_{triaz}$, **Cu-T**$_{tetraz}$, Cu foil, Cu_2O and CuO.

X-ray absorption fine structure (XAFS) spectra at Cu K-edge (E_0 = 8979 eV) were performed at BL14W1 beam line of Shanghai Synchrotron Radiation Facility (SSRF) operated at 3.5 GeV under "top-up" mode with a constant current of 240 mA. The energy was calibrated accordingly to the absorption edge of pure Cu foil. The Fourier transformed (FT) data in *R* space were analyzed by applying the 1st shell approximation model for the Cu-O and Cu-Cu for the extended X-ray absorption fine structure (EXAFS) part. For the X-ray absorption near edge structure (XANES) part, the experimental absorption coefficients as function of energies $\mu(E)$ were processed by background subtraction and normalization procedures, and reported as "normalized absorption" to compare with those of standard materials, Cu foil (Cu^0). The parameters describing the electronic properties (e.g., correction to the photoelectron energy origin, E_0) and local structure environment including coordination number (*CN*), bond distance (*R*) and Debye Waller factor around the absorbing atoms were allowed to vary during the fit process.

Table S2 Fitted structural parameters of T$_{imi}$-Cu, T$_{triaz}$-Cu and T$_{tetraz}$-Cu analyzed by using Cu K-edge (8979 eV) EXAFS. Amp: 0.90 ± 0.04 (Cu foil).

Sample	Cu-O		Cu-Cu		D. W.	ΔE_0/eV
	R/Å	CN	*R*/Å	CN		
Cu foil	—	—	2.542±0.003	12	0.0087±0.0003	4.6±0.5
Cu-T$_{imi}$	1.97±0.01	4.1±0.2	—	—	0.0055±0.0004	0.2±0.7
Cu-	1.97±0.	4.2±0	—	—		-

T_{treraz}	01	.2				0.1±0.5
Cu-T_{triaz}	1.95±0.01	4.5±0.3	—	—		-2.0±1.4

R, bond distance; CN, coordination number; D. W., Debye-Waller factor; ΔE_0, inner potential correction to account for the difference in the inner potential between the sample and the reference compound.

S2. Pore robustness and thermal stability

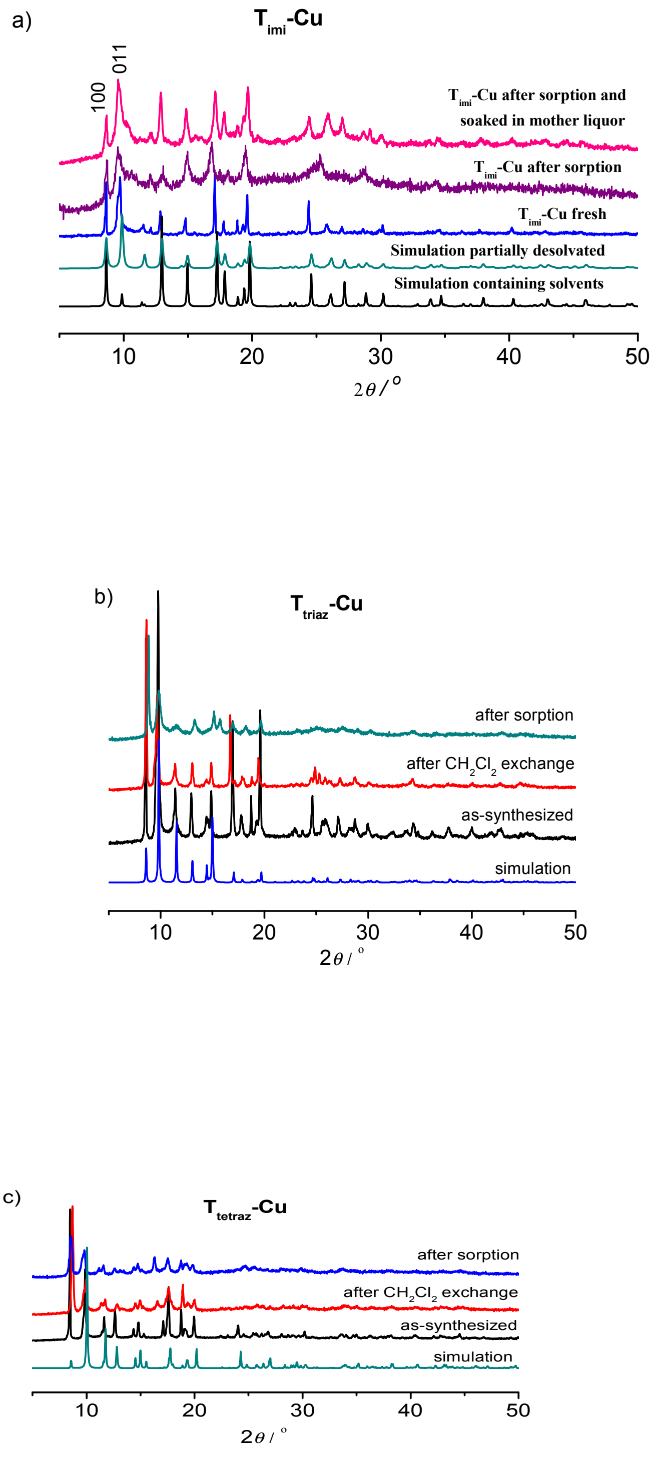

Figure S4 PXRD patterns of a) **T$_{imi}$-Cu** simulated from single crystal data, as-synthesized fresh sample, samples after sorption and recovered by soaking in mother liquor, and b) **T$_{triaz}$-Cu** and c) **T$_{tetraz}$-Cu** simulated from single crystal data, as-synthesized sample, samples soaked in CH_2Cl_2 for 3 d and after sorption measurements.

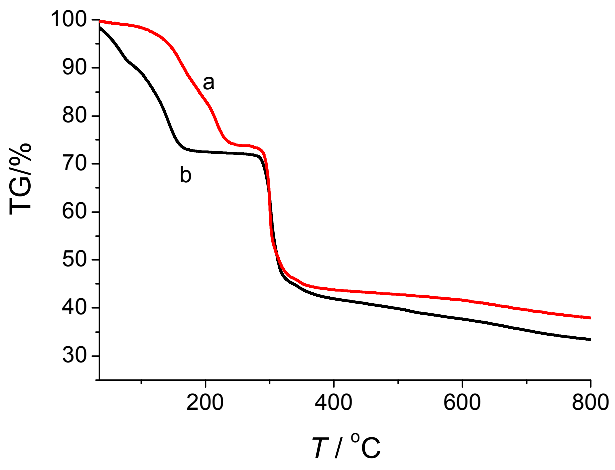

Figure S5 TGA curves for **T$_{imi}$-Cu**, a) as-synthesized and b) after CH_2Cl_2 exchange.

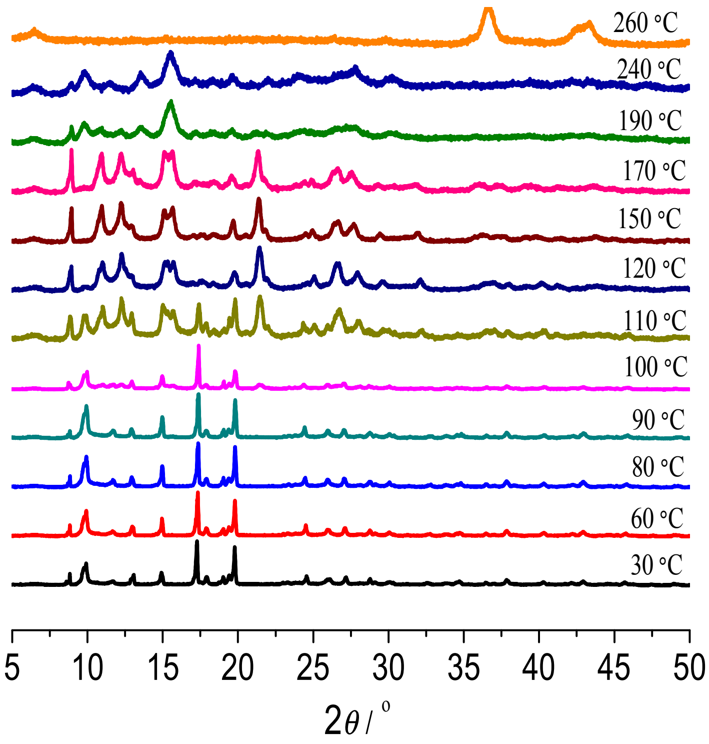

Figure S6 Temperature-dependent X-ray powder diffraction patterns for **T$_{imi}$-Cu**.

S3. Sorption study

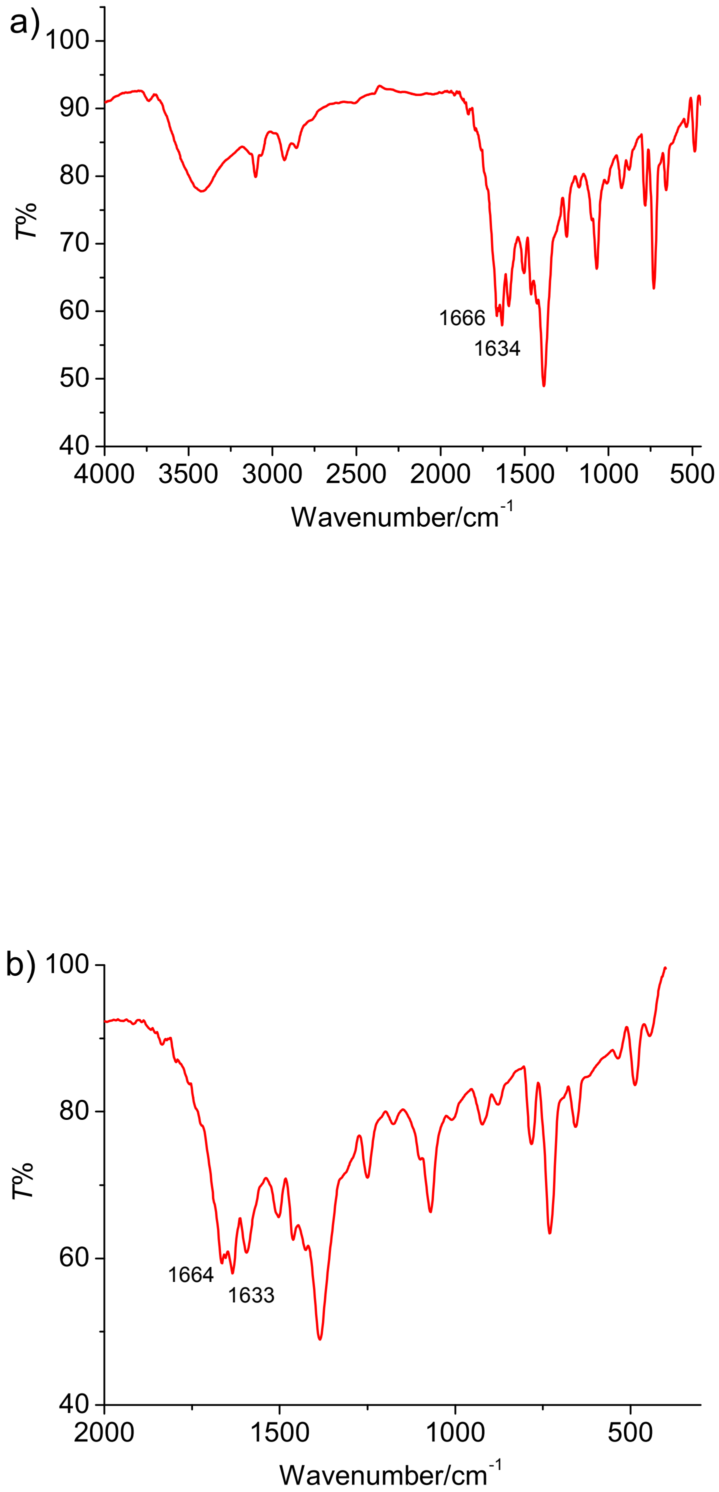

Figure S7 FT-IR spectra for $\mathbf{T_{imi}}$**-Cu**: a) as-synthesized and b) after desolvation.

Table S3 Activation method, surface area, pore size for **T$_{imi}$-Cu**, **T$_{triaz}$-Cu** and **T$_{tetraz}$-Cu**.

Materials	Activation method[a]	S_{BET} / m^2 g^{-1}	$S_{Langmuir}$ / m^2 g^{-1}	Pore volume /cm^3 g^{-1}	Pore size /Å
**T$_{imi}$-Cu**	i	771	1145	0.31	5.1, 6.8
**T$_{imi}$-Cu'**	ii	902	1444	0.37	5.1, 6.8
**T$_{triaz}$-Cu**	ii	768	893	0.29	4.9
**T$_{tetraz}$-Cu**	ii	766	1055	0.30	4.9

[a] Activation method: i, thermally activated under vacuum at 85 ºC; ii, thermally activated under vacuum at 85 ºC following CH_2Cl_2 solvent exchange. **T$_{imi}$-Cu** was chosen for detailed investigation due to its stability.

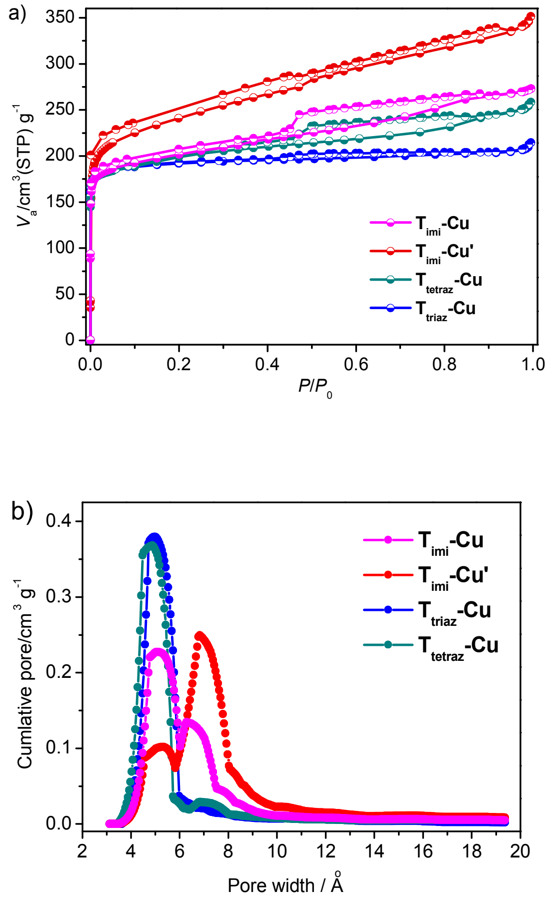

Figure S8 a) N_2 adsorption-desorption isotherms for **T$_{imi}$-Cu, T$_{imi}$-Cu', T$_{triaz}$-Cu** and **T$_{tetraz}$-Cu** measured at 77 K and b) Horvath-Kawazoe micropore size distribution.

H_2 sorption isotherms measured at 77 and 87 K indicate that H_2 uptake values at 77 K and 1 bar are relatively comparable, slightly varying in the sequence **T_{tetraz}-Cu** (8.4 mmol g^{-1}, 1.68 wt%) > **T_{triaz}-Cu** (8.0 mmol g^{-1}, 1.61 wt%) > **T_{imi}-Cu** (7.7 mmol g^{-1}, 1.55 wt%). The amounts of CH_4 uptake at 195 K change in similar order of **T_{tetraz}-Cu** ≈ **T_{triaz}-Cu** > **T_{imi}-Cu**. For **T_{tetraz}-Cu** and **T_{triaz}-Cu**, CH_4 uptake reaches 5.1 mmol g^{-1} (8.2 wt%), while that of **T_{imi}-Cu** (4.3 mmol g^{-1}, 7.0 wt%) decreases. The isosteric heats (Q_{st}) of H_2 sorption are calculated using the virial-type equation, falling in the range 5.2- 13.9 kJ mol^{-1}. Surprisingly, **T_{imi}-Cu** displays the largest H_2 sorption Q_{st}, which is estimated to be 13.9 kJ mol^{-1} at zero coverage. This value surpasses the values of many reported MOFs (5 ~ 9 kJ mol^{-1}) including those with open metal centers (e.g. 12.9 kJ mol^{-1} for Ni_2(dhtp) (Zhou *et al.* 2008) and 13.5 kJ mol^{-1} for COP-27-Ni[5]).

Zhou, W., Wu, H. & Yildirim, T. (2008) *J. Am. Chem. Soc.* **130**, 15268–15269.
Vitillo, J. G. *et al.* (2008) *J. Am. Chem. Soc.* **130**, 8386–8396.

a)

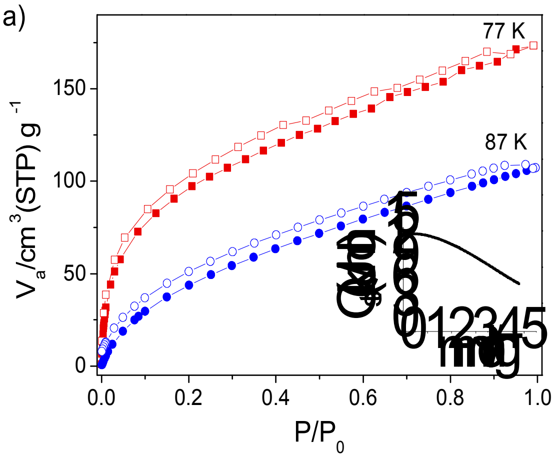
77 K
87 K
150
100
50
0
0.0
0.2
0.4
0.6
0.8
1.0
$V_a/cm^3(STP)\ g^{-1}$
P/P_0

b)

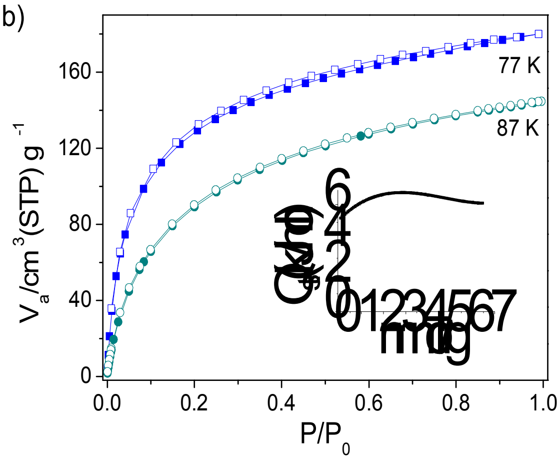
77 K
87 K
160
120
80
40
0
0.0
0.2
0.4
0.6
0.8
1.0
$V_a/cm^3(STP)\ g^{-1}$
P/P_0

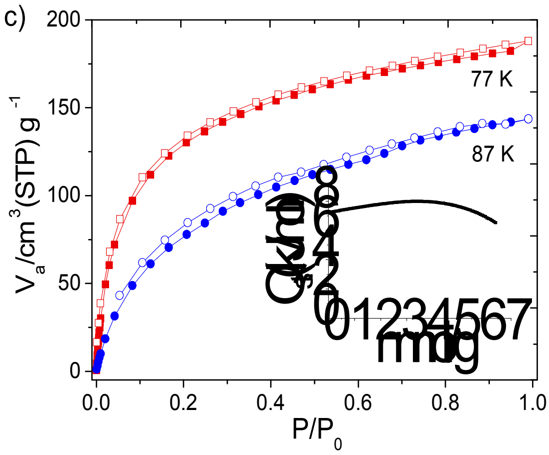

Figure S9 H_2 adsorption-desorption isotherm for a) **T$_{imi}$-Cu**, b) **T$_{triaz}$-Cu** and c) **T$_{tetraz}$-Cu** measured at 77 and 87 K with adsorption enthalpies are shown inset.

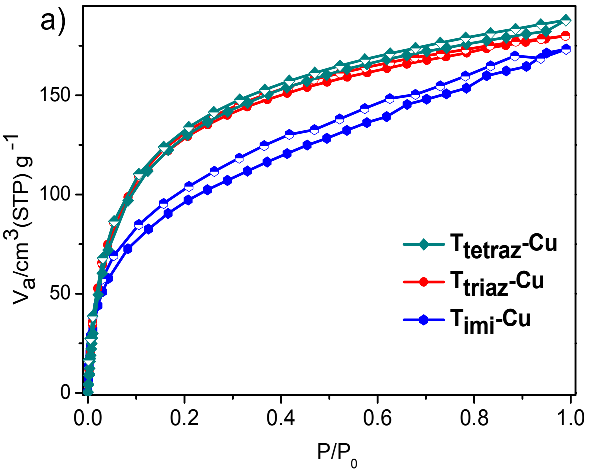

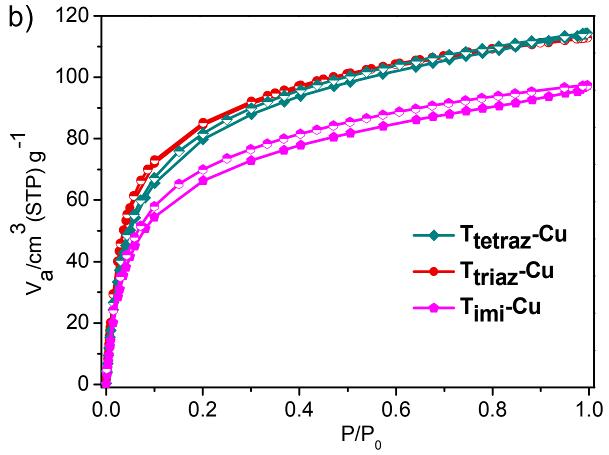

Figure S10 a) H_2 adsorption-desorption isotherms measured at 77 K and b) CH_4 adsorption-desorption isotherms measured at 195 K for **T$_{imi}$-Cu, T$_{triaz}$-Cu** and **T$_{tetraz}$-Cu**.

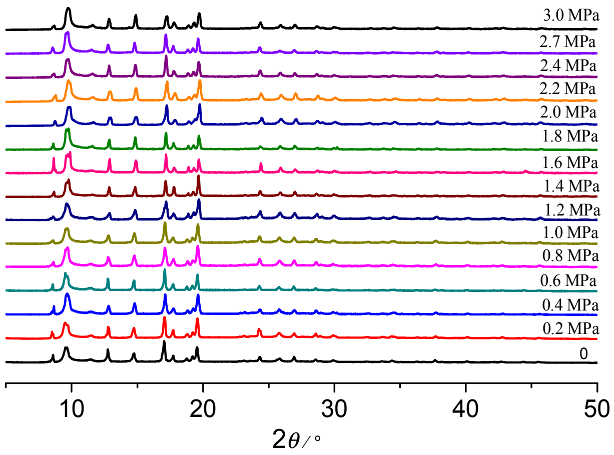

Figure S11 PXRD patterns of **T_{imi}-Cu** under high pressure CO_2 atmosphere from 0 to 3.0 MPa. The CO_2 high pressure was generated using a BELSORP-VC equipment, and the highest pressure of the equipment is 3.4 MPa.

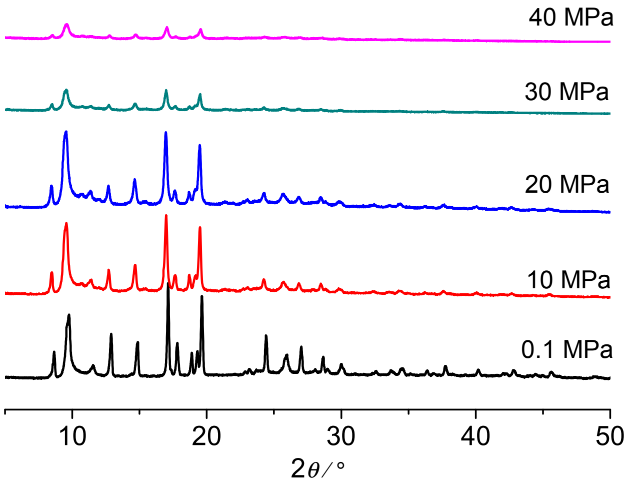

Figure S12 PXRD patterns of **T_{imi}-Cu** tablet compressed with a tablet press with the pressure from 0 to 40 MPa.

S4. Sorption simulation

1) Model preparation. The models were all optimized with the Dmol3 model of Materials Studio 6.1 (Accelrys, Materials Studio Release Notes, Release 6.1.0, Accelrys Software, Inc.: San Diego, 2012). Quality is Fine. Functional is GGA, PBE. Basis set is DNP 4.4. Population analysis was selected. Models without DMF molecules were chosen. The DMF molecules and disorder atoms in the crystal structure were rationally removed, then all the atoms were fixed and only the H atoms were optimized. Then a model was obtained namely conformation **1**. Then the imidazole ring was slightly rotated. All atoms except H atoms and the imidazole ring were fixed. The structure was optimized and named as conformation **2.** The imidazole ring is rotated for 5.448^{o}.

2) Sorption simulation. The sorption module of Materials Studio 6.1 was used to simulate CO_2 adsorption at 263 K from 0.1 kPa to 100 kPa. a) CO_2 sorbate preparation. A carbon dioxide molecule is geometrically optimized with DMol3 module. Quality is fine; Functional is B3LYP. Basis set is DNP-4.4. Population analysis is calculated. Then the charge distribution is applied to the N_2 model. b) Adsorption isotherm calculation. Method is Metropolis. Quality is ultra-fine. Fugacity range is 0.1-100 kPa with 20 steps. Temperature is 263 K. Charges is Charge-using-QEq. Force field is Universal.

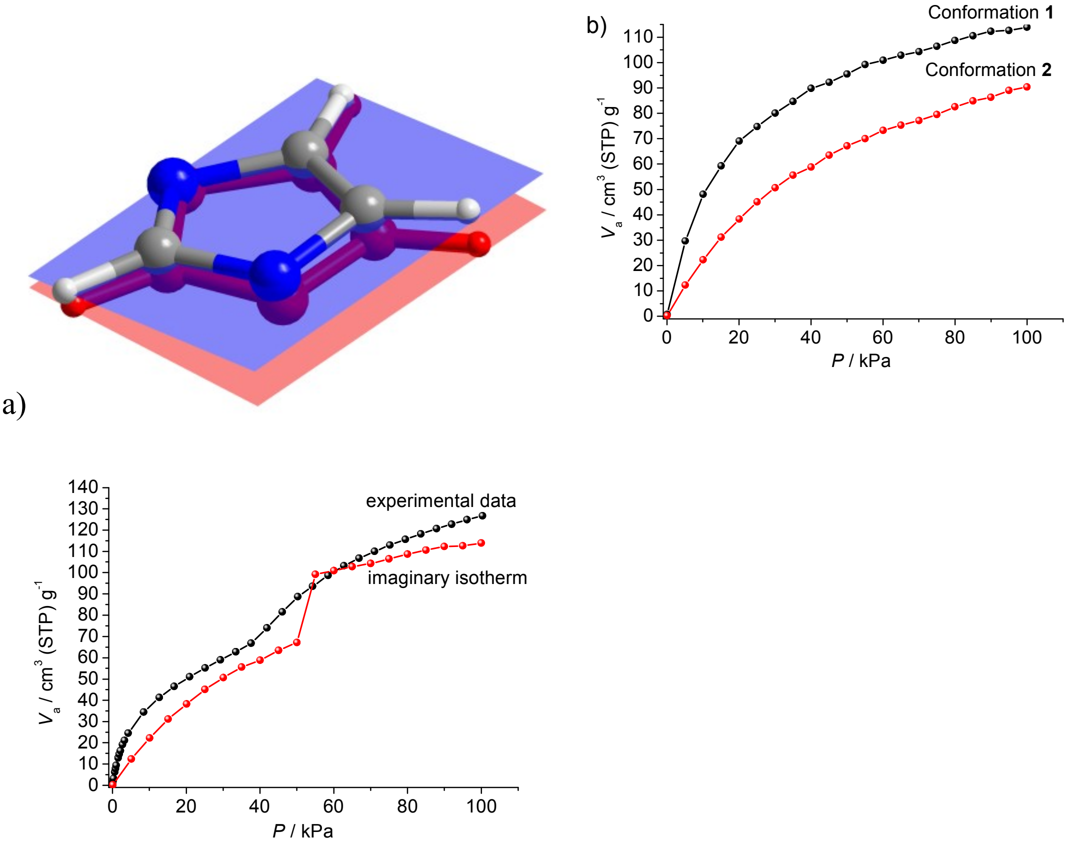

Figure S13 a) Twist of the imidazole ring, blue/red plane labelled conformation **1**/**2**, respectively; b) simulated CO_2 adsorption isotherm under 263 K; c) experimental data and imaginary isotherm of CO_2 uptake at 263 K. The imaginary stepwise isotherm is obtained by assuming the ligand adopt conformation **2**/**1** before/after 50 kPa.
